# Dissecting the genetic basis of wheat blast resistance in the Brazilian wheat cultivar BR 18-Terena

**DOI:** 10.1186/s12870-020-02592-0

**Published:** 2020-08-27

**Authors:** Rachel Goddard, Andrew Steed, Catherine Chinoy, Jéssica Rosset Ferreira, Pedro Luiz Scheeren, João Leodato Nunes Maciel, Eduardo Caierão, Gisele Abigail Montan Torres, Luciano Consoli, Flavio Martins Santana, José Mauricio Cunha Fernandes, James Simmonds, Cristobal Uauy, James Cockram, Paul Nicholson

**Affiliations:** 1 Department of Crop Genetics, John Innes Centre, Norwich Research Park, Norwich, UK; 2 Universidade de Passo Fundo, Passo Fundo, RS Brazil; 3 Embrapa Trigo, Passo Fundo, RS Brazil; 4 John Bingham Laboratory, NIAB, Cambridge, UK

**Keywords:** Wheat blast, *Magnaporthe oryzae*, Quantitative trait loci, Seedling resistance, Head resistance, Single nucleotide polymorphism (SNP) genotyping, *Triticum aestivum*

## Abstract

**Background:**

Wheat blast, caused by *Magnaporthe oryzae Triticum* (MoT) pathotype, is a global threat to wheat (*Triticum aestivum* L.) production. Few blast resistance (*R*) genes have been identified to date, therefore assessing potential sources of resistance in wheat is important. The Brazilian wheat cultivar BR 18-Terena is considered one of the best sources of resistance to blast and has been widely used in Brazilian breeding programmes, however the underlying genetics of this resistance are unknown.

**Results:**

BR 18-Terena was used as the common parent in the development of two recombinant inbred line (RIL) F_6_ populations with the Brazilian cultivars Anahuac 75 and BRS 179. Populations were phenotyped for resistance at the seedling and heading stage using the sequenced MoT isolate BR32, with transgressive segregation being observed. Genetic maps containing 1779 and 1318 markers, were produced for the Anahuac 75 × BR 18-Terena and BR 18-Terena × BRS 179 populations, respectively. Five quantitative trait loci (QTL) associated with seedling resistance, on chromosomes 2B, 4B (2 QTL), 5A and 6A, were identified, as were four QTL associated with heading stage resistance (1A, 2B, 4A and 5A). Seedling and heading stage QTL did not co-locate, despite a significant positive correlation between these traits, indicating that resistance at these developmental stages is likely to be controlled by different genes. BR 18-Terena provided the resistant allele for six QTL, at both developmental stages, with the largest phenotypic effect conferred by a QTL being 24.8% suggesting that BR 18-Terena possesses quantitative resistance. Haplotype analysis of 100 Brazilian wheat cultivars indicates that 11.0% of cultivars already possess a BR 18-Terena-like haplotype for more than one of the identified heading stage QTL.

**Conclusions:**

This study suggests that BR 18-Terena possesses quantitative resistance to wheat blast, with nine QTL associated with resistance at either the seedling or heading stage being detected. Wheat blast resistance is also largely tissue-specific. Identification of durable quantitative resistances which can be combined with race-specific *R* gene-mediated resistance is critical to effectively control wheat blast. Collectively, this work facilitates marker-assisted selection to develop new varieties for cultivation in regions at risk from this emerging disease.

## Background

Wheat blast, or brusone, is caused by the fungal pathogen *Magnaporthe oryzae Triticum* (MoT) pathotype (synonym *Pyricularia oryzae*) and is a potential global threat to wheat production. The most commonly observed symptom of wheat blast is the complete or partial bleaching of infected wheat heads, but the disease may also manifest as eye-shaped, grey lesions on the leaves and stems of wheat plants [1]. Wheat blast was first identified in Paraná, Brazil in 1985 [2], where it then spread throughout the wheat growing areas of South America, reaching Bolivia in 1996 and Paraguay and Argentina by 2007 [3]. The disease remained confined to South America until 2016, when a severe outbreak of wheat blast emerged in Bangladesh [4]. The presence of blast in South Asia leads to concerns that wheat production in India, the world’s second largest wheat producer, may be seriously affected. In Brazil, blast has been a major limiting factor of wheat production for decades, particularly in the central Cerrado region where the humid, sub-tropical climate provides an ideal environment for disease development [3]. As wheat blast has the potential to cause up to 100% crop losses, it is critical to develop disease management strategies to benefit both the regions where blast is endemic and those where it is a newly emerging threat. Chemical control measures have proven unreliable in the management of wheat blast [5, 6] and resistance to strobilurin and triazole fungicides has already been observed in Brazil [7, 8]. It is therefore essential to identify reliable genetic resistances to adequately control disease.

Whilst the *M. oryzae* species complex is able to cause blast disease on over 50 grass species, host-adapted lineages are observed within the species complex [4]. Isolates infecting wheat (*Triticum MoT* pathotype), rice (*Oryza MoO* pathotype), turf grass (*Lolium* pathotype), finger millet (*Eleusine* pathotype) and foxtail millet (*Setaria* pathotype) form genetically distinct groups following phylogenomic analysis [4]. Pathotypes also show limited pathogenicity on alternative hosts [1, 9]. As with rice blast, wheat blast resistance is thought to be governed by specific gene-for-gene interactions between host resistance (*R*) genes and race-specific avirulence (*AVR*) genes within the pathogen [10]. At present, few resistance genes have been identified in wheat. *Rmg2* (*Resistance to Magnaporthe grisea 2*) (chromosome 7A) and *Rmg3* (6B), identified in the hexaploid wheat (*Triticum aestivum*) cultivar Thatcher, confer blast resistance at the seedling stage and are temperature sensitive [11]. The genes *Rmg7* (2A) and *Rmg8* (2B), from tetraploid (*T. durum*) and hexaploid wheat respectively, recognise the same avirulence gene *AVR-Rmg8* and provide resistance at both the seedling and heading stage [12]. However, of the two *R* genes only *Rmg8* has been determined to be effective at temperatures above 24 °C [12]. The genes *Rmg1* (syn. *Rwt4*) (1D) and *Rmg6* (syn. *Rwt3*) (1D) also provide resistance in both seedlings and heads of wheat [13], however *Rmg6* is temperature sensitive and is ineffective above 25 °C [14]. Unfortunately, reports already suggest that *Rmg2, Rmg3* and *Rmg7* have been overcome by more aggressive field MoT isolates [1], indicating the importance of identifying additional sources of resistance. The 2NS/2AS chromosomal translocation originating from the wheat wild relative *Aegilops ventricosa* has been shown to confer wheat blast resistance at the heading stage, with cultivars carrying the 2NS translocation displaying up to a 72% reduction in disease symptoms compared to those without 2NS [15]. Resistance conferred by the 2NS translocation has, however, also been demonstrated to be less effective to recent blast isolates [15]. It is also ineffective in certain genetic backgrounds [16], suggesting the presence of the 2NS translocation cannot be solely relied upon to provide adequate resistance.

Several studies have revealed contrasting blast resistance responses at different wheat development stages, such as with the 2NS translocation which confers resistance in the head but has no effect on foliar resistance [15]. A study of 85 U.S. wheat cultivars demonstrated that resistance to blast at the seedling stage may not be a reliable indicator of resistance at the heading stage [17]. A similar observation was also made by Martinez et al. [18] who found a low negative correlation between disease severity at the seedling and heading stage in Argentinian wheat cultivars. Whilst such studies demonstrate varietal differences in response to seedling and head infection, little is known at the genetic level. A more thorough understanding of the genetics controlling blast resistance is therefore essential to provide continued resistance throughout the different stages of wheat development.

In rice [*Oryza sativa*] over 100 blast quantitative trait loci (QTL) have been identified and 35 major *R* genes have been cloned and molecularly characterised [19], suggesting that within the rice gene pool there is considerable natural variation associated with blast resistance. The barley gene pool may also be a rich source of *M. oryzae* resistance, with 9.0% of European barley cultivars tested by Aghnoum et al. [20] showing complete resistance to both MoT and MoO pathotypes. In wheat, only nine *R* genes (*Rmg1* – *Rmg8*, *RmgGR119*) have been identified to date [21], and very few blast QTL have been detected, which may indicate a more limited gene pool for resistance. As such, it is particularly important to thoroughly assess any potential sources of moderate to high resistance in wheat. The Brazilian wheat cultivars BR 18-Terena, BRS 229 and MGS3 Brilhante have been shown to display consistent, moderate resistance to blast both under field and controlled conditions [6, 22, 23], suggesting they possess valuable resistance. In particular, BR 18-Terena displayed broad spectrum seedling resistance when inoculated with 72 blast isolates by Urashima et al. [22], and also moderate resistance when inoculated with 69 isolates at the seedling stage and 27 isolates at the heading stage by Maciel et al. [24]. Due to this consistent, moderate resistance, BR 18-Terena has been frequently used in Brazilian breeding programmes since its release in 1986 [25], and many Brazilian wheat cultivars contain BR 18-Terena within their pedigree [26]. However, whilst the durable resistance of BR 18-Terena has been widely utilised, the underlying genetics of this resistance are still unknown. Understanding whether the resistance of BR 18-Terena is due to quantitative or *R* gene-mediated resistance is particularly important for wheat breeders, as is identifying the developmental stages protected by the resistances.

The aim of this study was to gain the first insight into the genetic basis of wheat blast resistance in BR 18-Terena with the aim of identifying specific resistances which may be advantageous for use in wheat breeding programmes. BR 18-Terena, hereafter referred to as BR 18, was used as the common parent in two bi-parental crosses developed with the Brazilian wheat cultivars Anahuac 75 and BRS 179. The two recombinant inbred line (RIL) populations were phenotyped for resistance at both the seedling and heading stage using the sequenced MoT isolate BR32 [27]. The populations were genotyped using the Axiom® 35 k Wheat Breeders’ Array [28] and genetic linkage maps were generated for each population, to allow the identification of QTL associated with wheat blast resistance.

## Results

### Blast phenotyping

**Fig. 1:**
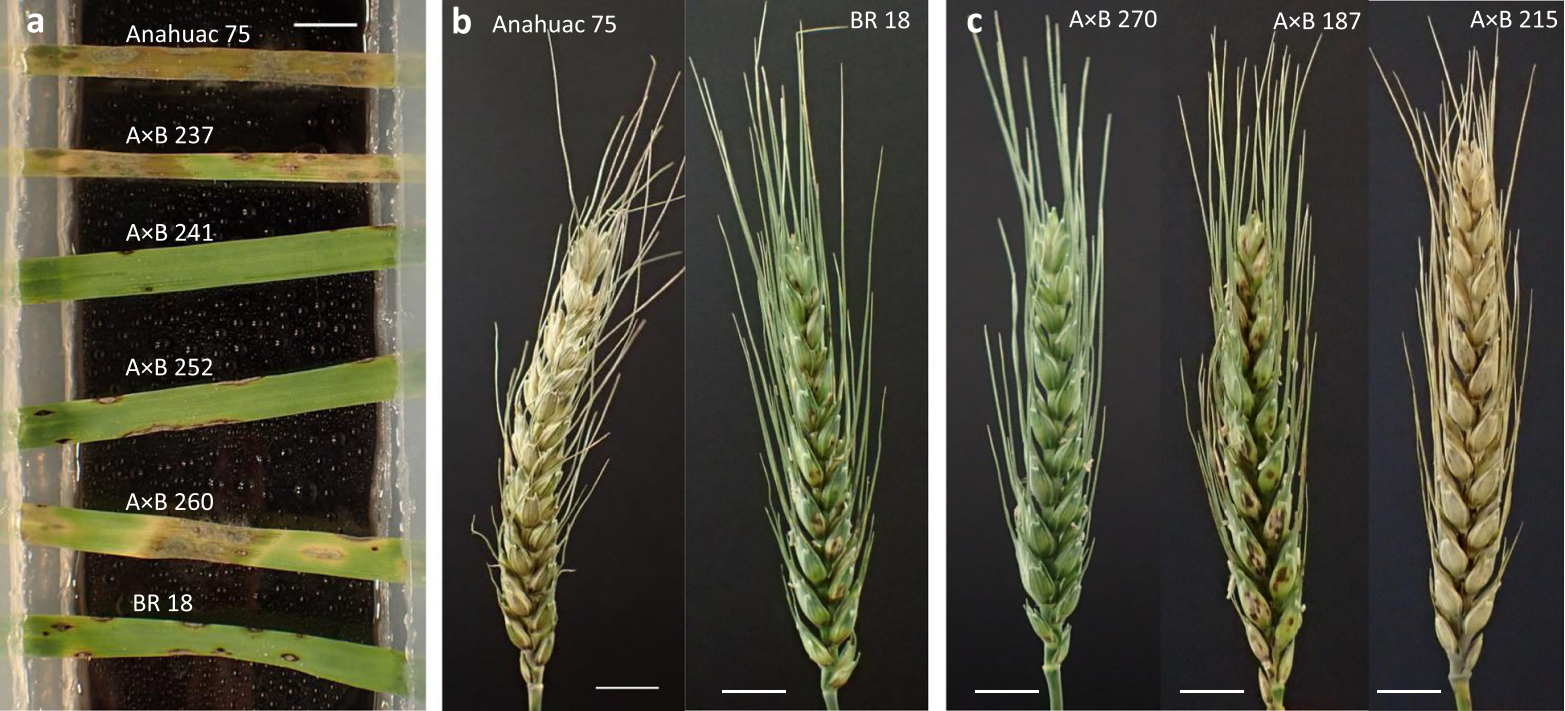
Wheat blast assays in the Anahuac 75 × BR 18 population. **a** Detached leaf assay symptoms with Anahuac 75 × BR 18 F_6_ RILs inoculated with MoT BR32 isolate at 6 dpi. **b** Detached head assay with Anahuac 75 and BR 18 inoculated with MoT BR32 isolate at 9 dpi. **c** Detached head assay with F_6_ RILs inoculated with MoT BR32 isolate at 9 dpi. Scale bar = 1 cm

**Fig. 2:**
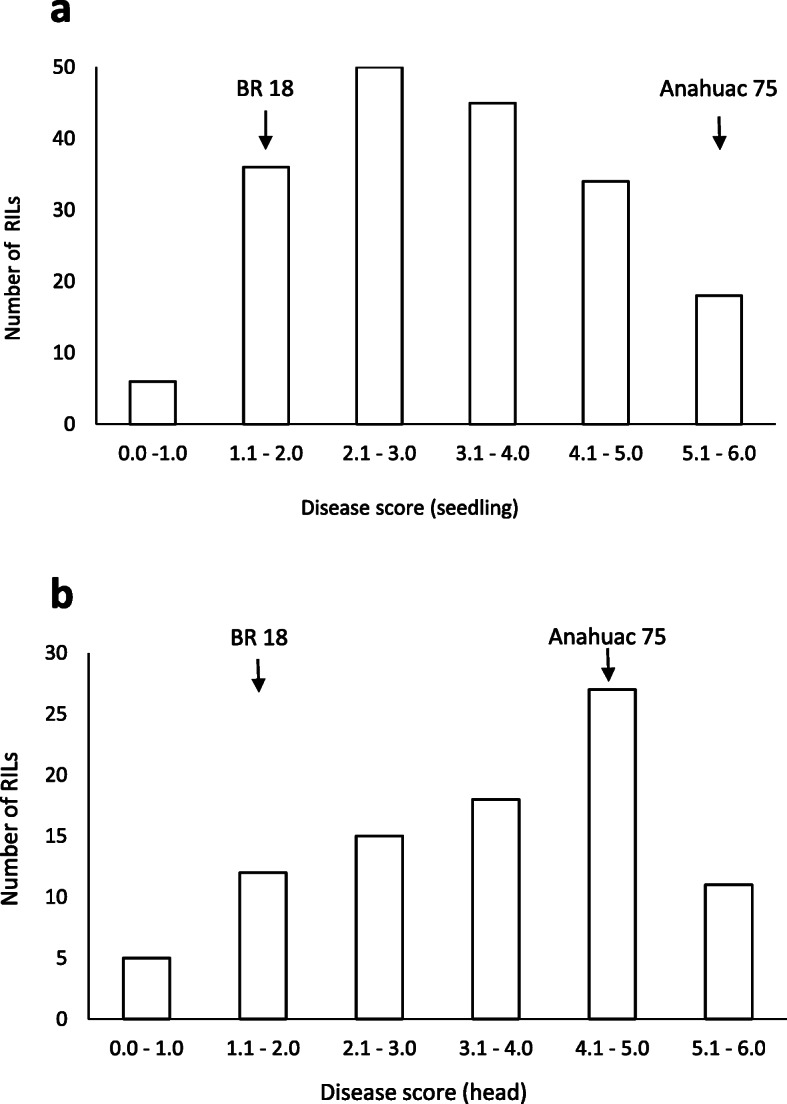
Phenotypic distributions in the Anahuac 75 × BR 18 F_6_ RIL population. **a** Predicted mean disease scores for resistance at the seedling stage. **b** Predicted mean disease scores for resistance at the heading stage. Arrows indicate the indicate the predicted mean scores of Anahuac 75 and BR 18 within each distribution

**Fig. 3:**
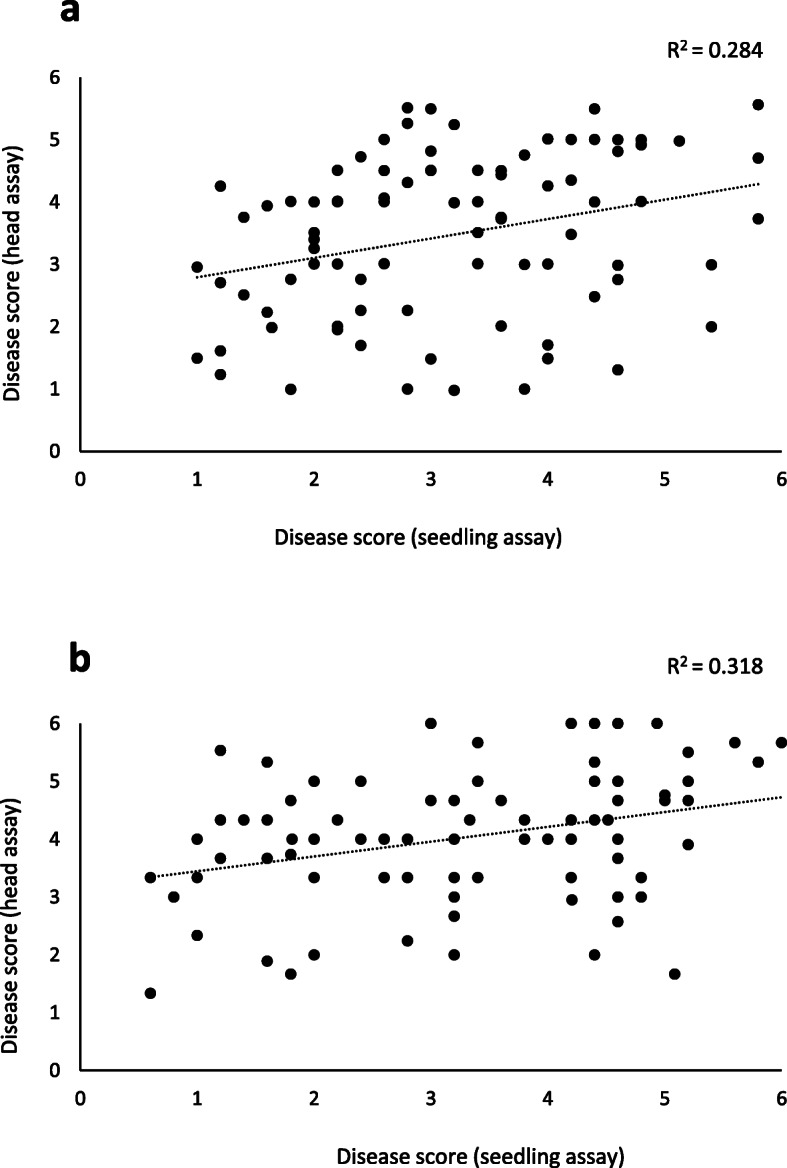
Phenotypic correlations between mean disease scores. **a** The correlation between predicted mean disease scores for the seedling and head assays in the Anahuac 75 × BR 18 F_6_ RILs. **b** The correlation between predicted mean disease scores for the seedling and head assays in the BR 18 × BRS 179 F_6_ RILs

In the seedling assays for blast susceptibility, Anahuac 75 had a mean score of 5.1 (Table 1), displaying water soaking and grey sporulating lesions which indicate complete susceptibility of the leaf tissue (Fig. 1a). In contrast, the leaves from BR 18 remained green with small grey lesions ringed with brown necrosis (disease score 1.6). Significant differences (*P* < 0.001) between the predicted mean scores for Anahuac 75 and BR 18 for the seedling assays were observed (Table 1). In the Anahuac 75 × BR 18 RILs, the mean seedling score was 3.2, with a range of 0.8–6.0, suggesting transgressive segregation (Table 1). In the detached head assays, Anahuac 75 displayed severe bleaching of the head (mean score 5.0) whilst BR 18, with a mean disease score of 2.0, remained green with some minor bleaching and necrosis (Fig. 1b). The differences in the predicted mean scores between the parental lines for the detached head assay were significant at the *P* < 0.001 level (Table 1). In the Anahuac 75 × BR 18 RILs, the mean head disease score was 3.5, with a range of 0.9–6.0, again suggesting transgressive segregation in the population. The frequency distribution of disease scores for both the detached leaf and detached head assays are shown in Fig. 2. A significant positive correlation (R^2^ = 0.284, *P* = 0.007) between the predicted mean scores for the seedling and detached head experiments was observed, as shown in Fig. 3a.

**Table 1:** Mean disease scores of Anahuac 75, BR 18 and the Anahuac 75 × BR 18 RILs

Growth stage	Mean disease score		t- prob^a^	RILs	
	Anahuac 75	BR 18		Mean	Range
Seedling	5.1	1.6	< 0.001	3.2	0.8–6.0
Head	5.0	2.0	< 0.001	3.5	0.9–6.0

^a^The statistical significance of the difference between predicted mean scores for Anahuac 75 and BR 18 are shown by t-probabilities calculated within the GLM

**Fig. 4:**
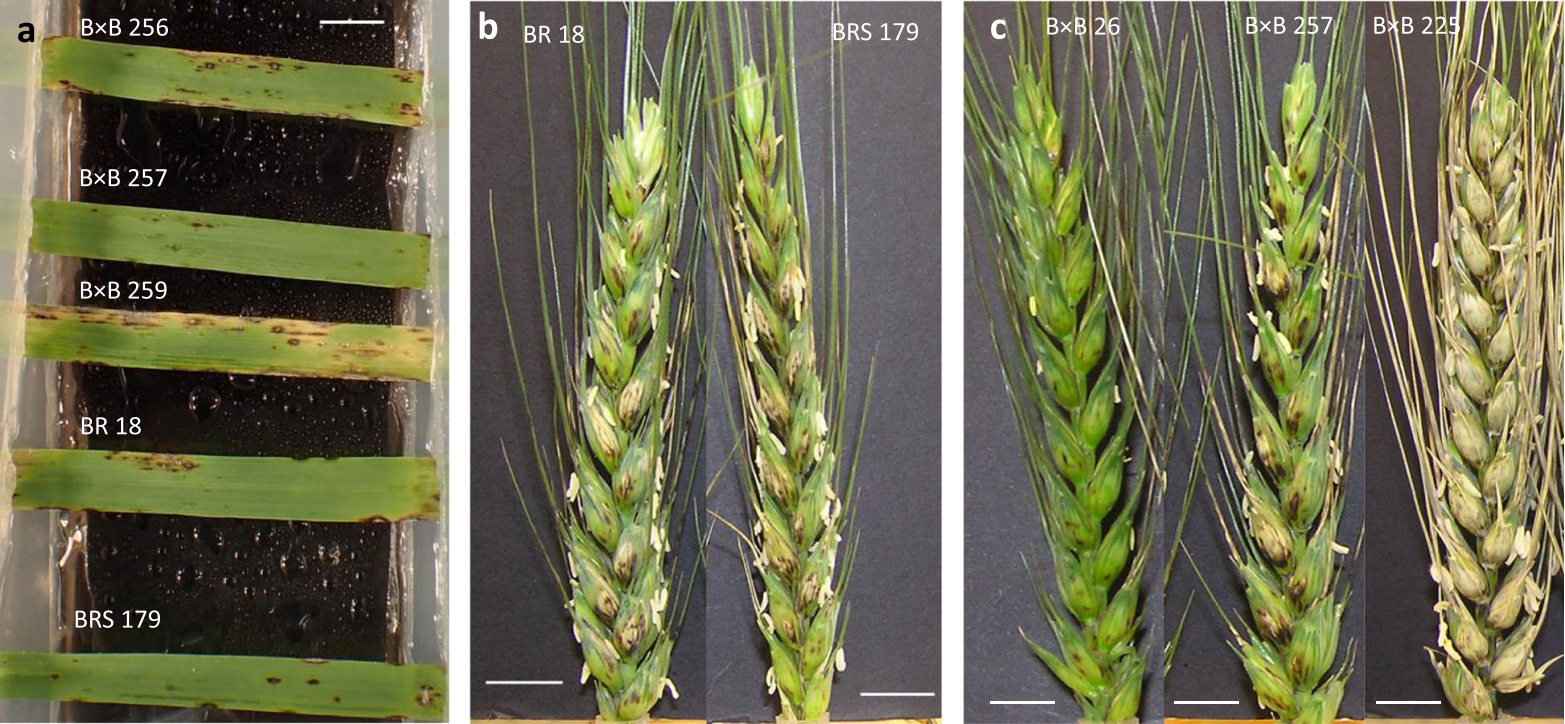
Wheat blast assays in the BR 18 × BRS 179 population. **a** Detached leaf assay symptoms with BR 18 × BRS 179 F_6_ RILs inoculated with MoT BR32 isolate at 6 dpi. **b** Detached head assay with BR 18 and BRS 179 inoculated with MoT BR32 isolate at 9 dpi. **c** Detached head assay with F_6_ RILs inoculated with MoT BR32 isolate at 9 dpi. Scale bar = 1 cm

**Fig. 5:**
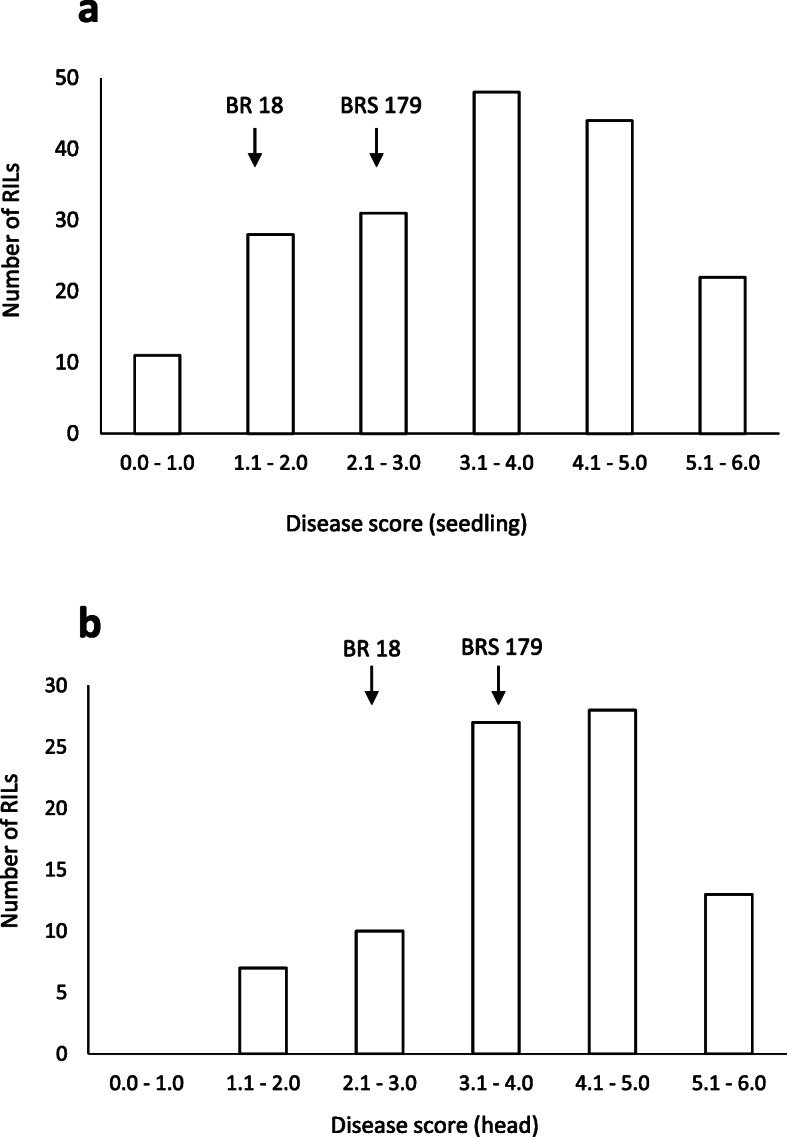
Phenotypic distributions in the BR 18 × BRS 179 F_6_ RIL population. **a** Predicted mean disease scores for resistance at the seedling stage. **b** Predicted mean disease scores for resistance at the heading stage. Arrows indicate the indicate the predicted mean scores of BR 18 and BRS 179 within each distribution

BR 18 and BRS 179 both displayed similar symptoms following seedling inoculation, with small necrotic lesions and small grey lesions ringed with necrosis (Fig. 4a). There was no significant difference between the predicted mean scores of BR 18 and BRS 179, which were 1.8 and 2.7, respectively (Table 2). Within the BR 18 × BRS 179 RIL population, the range of seedling scores was 0.4–6.0 indicating transgressive segregation, with a mean of 3.4 (Table 2). As with the seedling assay, there was little difference in susceptibility between BR 18 and BRS 179 at the heading stage. The mean scores were 3.0 and 4.0, for BR 18 and BRS 179 respectively, indicating necrotic lesions and minor bleaching of the glumes (Fig. 4b). These scores were not significantly different (*P =* 0.346) (Table 2). A mean disease score of 4.0 was observed in the BR 18 × BRS 179 RILs, with a range of 1.3–6.0, indicating transgressive segregation (Table 2). Histograms showing the frequency distribution of disease scores for both the detached leaf and detached head assays are shown in Fig. 5. A significant positive correlation (R^2^ = 0.318, *P =* 0.003) between the predicted mean scores for the seedling and head experiments was observed, as shown in Fig. 3b.

**Table 2:** Mean disease scores of BR 18, BRS 179 and the BR 18 × BRS 179 RILs

Growth stage	Mean disease score		t- prob^a^	RILs	
	BR 18	BRS 179		Mean	Range
Seedling	1.8	2.6	0.258	3.4	0.4–6.0
Head	3.0	4.0	0.346	4.0	1.3–6.0

^a^The statistical significance of the difference between predicted mean scores for BR 18 and BRS 179 are shown by t-probabilities calculated within the GLM

### Genetic mapping

In the Anahuac 75 × BR 18 population, a total of 3641 markers were polymorphic between Anahuac 75 and BR 18 and were suitable for genetic mapping. The mean residual heterozygosity for co-dominant markers was determined to be 3.3%, with the expected heterozygosity for an F_6_ generation being 3.1%. A total of 3180 markers were assigned to a linkage group, of which 1779 markers had unique genetic map positions (Table 3). The final genetic linkage map covered 3933.6 cM across the 21 wheat nuclear chromosomes, giving a marker coverage of one marker every 2.2 cM. The A genome was represented by 767 markers, the B genome by 739 markers and the D genome by 273 markers. The most marker dense linkage group was chromosome 1A, with 174 markers, whilst the most sparsely populated linkage group was 6D, with 16 markers.

**Table 3:** Marker distribution in the Anahuac 75 × BR 18 F_6_ genetic linkage map

Chr.^a^	Number of markers	Length (cM)
1A	174	319.1
1B	105	192.5
1D	135	297.9
2A	115	293.2
2B	140	251.9
2D	24	120.4
3A	137	265.5
3B	132	244.8
3D	24	153.8
4A	98	270.3
4B	102	192.2
4D	20	77.9
5A	95	221.5
5B	146	260.0
5D	29	59.9
6A	102	273.0
6B	28	45.4
6D	16	30.9
7A	46	87.7
7B	86	225.4
7D	25	50.3
	1779	3933.6

^a^*Chr* chromosome

In the BR 18 × BRS 179 population, there were 4110 polymorphic markers which were suitable for genetic mapping and the mean residual heterozygosity for co-dominant markers was 2.5%. A total of 3096 markers were assigned to a linkage group, of which 1318 markers had a unique map position (Table 4). The final genetic linkage map covered 2856.6 cM, giving a marker coverage of one marker every 2.2 cM. The A genome was represented by 690 markers, the B genome by 556 markers and the D genome by 72 markers. The most marker dense linkage group was 3A, with 150 markers, whilst the most sparsely populated linkage group was 7D, with only 4 markers.

**Table 4:** Marker distribution in the BR 18 × BRS 179 F_6_ genetic linkage map

Chr.^a^	Number of markers	Length (cM)
1A	106	221.9
1B	103	134.2
1D	25	68.4
2A	140	255.4
2B	77	130.1
2D	11	53.6
3A	150	346.1
3B	65	120.7
3D	7	38.7
4A	65	138.5
4B	86	169.1
4D	5	16.5
5A	115	269.6
5B	114	234
5D	6	21.1
6A	50	95.1
6B	59	151.1
6D	14	45.3
7A	64	197.6
7B	52	128
7D	4	21.6
	1318	2856.6

^a^Chr chromosome

### QTL mapping

In the Anahuac 75 × BR 18 population, two QTL were identified which were associated with seedling resistance to MoT BR32 (Table 5). A QTL on chromosome 4B explained 7.9% of the phenotypic variance, whilst a QTL on 6A explained up to 5.9% of the variance, with BR 18 conferring resistance at both of these loci (Table 5). Three QTL associated with head blast were identified. Two QTL with BR 18 conferring the resistant allele were identified on chromosomes 4A and 5A, explaining up to 17.8 and 18.8% of the phenotypic variance, respectively (Table 5). A single QTL with Anahuac 75 conferring resistance was identified on 1A, explaining up to 10.4% of the variance. QTL positions in the context of the genetic maps for the relevant chromosomes are shown in Additional Files 1, 2, 3, 4, and 5.

**Table 5:** Wheat blast QTL identified in the Anahuac 75 × BR 18 RIL population

QTL	Peak marker	Chr^*^	Position (cM)	QTL interval (cM)	LOD	% Var^*^	Additive effect	Low disease allele	s.e.
*QSdl.jic-4B.1*	AX-94926956	4B	129.4	103.5–155.3	4.0	7.9	0.4	BR 18	0.1
*QSdl.jic-6A*	AX-94812346	6A	103.1	53.4–148.8	3.2	5.9	0.3	BR 18	0.1
*QHead.jic-1A*	AX-94894053	1A	52.4	35.4–69.4	3.0	10.4	0.3	Anahuac 75	0.1
*QHead.jic-4A*	AX-94475087	4A	15.8	7.8–23.7	4.4	17.8	0.4	BR 18	0.1
*QHead.jic-5A*	AX-95229410	5A	216.5	206.5–221.5	5.0	18.8	0.6	BR 18	0.1

^a^*Chr* chromosome, % Var = percent of the phenotypic variation explained

In the BR 18 × BRS 179 population, three QTL associated with blast seedling resistance were identified (Table 6). Two QTL were identified on 4B and 5A, explaining up to 24.8 and 16.8% of the phenotypic variance respectively, with BRS 179 contributing the low disease allele at both loci (Table 6). An additional QTL on chromosome 2B explained up to 4.6% of the variance seen within the population, with BR 18 conferring the low disease allele. A single QTL associated with head blast resistance was identified on 2B, which explained up to 19.6% of the variance. At this locus, BR 18 conferred resistance (Table 6). QTL positions in the context of the genetic maps for the relevant chromosomes are shown in Additional Files 6, 7, and 8.

**Table 6:** Wheat blast QTL identified in the BR 18 × BRS 179 RIL population

QTL	Peak marker	Chr^a^	Position (cM)	QTL interval (cM)	LOD	% Var^a^	Additive effect	Low disease allele	s.e.
*QSdl.jic-2B*	AX-94797910	2B	83.8	0.0–130.0	3.5	4.6	0.3	BR 18	0.1
*QSdl.jic-4B.2*	AX-94812592	4B	127.4	120.9–133.8	13.3	24.8	0.6	BRS 179	0.1
*QSdl.jic-5A*	AX-94785956	5A	233.5	224.4–242.7	10.4	16.8	0.5	BRS 179	0.1
*QHead.jic-2B*	AX-94484517	2B	98.8	88.8–108.8	3.4	19.6	0.4	BR 18	0.1

^a^*Chr* chromosome, % Var = percent of the phenotypic variation explained

### Haplotype analysis of a Brazilian wheat cultivar panel

Four QTL were identified that were associated with blast resistance at the heading stage. Anahuac 75 conferred the resistant allele for the QTL on chromosome 1A (explaining 10.4% of the phenotypic variation, var) in the Anahuac 75 × BR 18 population. BR 18 conferred the resistant allele for QTL on chromosome 2B (19.6% var) in the BR 18 × BRS 179 population, and on 4A (17.8% var) and 5A (18.8% var) in the Anahuac 75 × BR 18 population. The haplotypes of a panel of 100 Brazilian wheat cultivars were compared to those of either BR 18 or Anahuac 75 at the respective QTL regions, with the aim of identifying cultivars possessing a similar haplotype (98.0% identical genotype calls) to the resistant parent within each QTL region (Additional File 9). The 1A QTL interval, which represents the physical region of 517,894,785 – 586,284,850 bp on 1A, was populated by 422 markers, as shown in Additional File 9. In the Brazilian wheat panel, 18.0% of the cultivars displayed a haplotype similar to Anahuac 75 within the 1A QTL region, as shown in Additional File 10. The 2B QTL interval (666,652,404 – 747,821,399 bp) was represented by 206 markers. A BR 18-like haplotype within the 2B region was observed in 54.0% of the Brazilian wheat cultivars (Additional File 10). The 4A QTL interval was represented by 73 markers and covered the smallest physical region (3,868,572 – 16,965,499 bp). Within this interval, 9.0% of the Brazilian wheat panel displayed a BR 18-like haplotype. The 5A QTL region (570,008,652 – 596,561,078 bp), was covered by 119 markers. A BR 18-like haplotype within this region was observed in 10.0% of the Brazilian wheat cultivars. In total, 16.0% of cultivars possessed a haplotype similar to the resistant parent (either BR 18 or Anahuac 75, depending on the loci) at two of the QTL regions identified, whilst 2.0% of cultivars possessed a haplotype similar to the resistant parent for three of the QTL regions (Additional File 10).

## Discussion

### Wheat blast resistance is developmental stage-specific

Wheat blast can affect the wheat plant from the early seedling stage until the late reproductive stage, in a comparable manner to blast observed on the leaves and panicles in rice [1]. Several studies have compared the resistance of wheat cultivars at both the vegetative and reproductive growth stages, however the results have been inconclusive as both positive and negative correlations between seedling and heading stage resistance have been observed [17, 18, 24]. In our study a significant positive correlation between seedling and head blast scores was observed in both populations, however the QTL associated with blast at the different developmental stages were not coincident. This is a similar observation to Cruz et al. [17], who saw a positive correlation between blast symptoms at the leaf and heading stage in a study of U.S. wheat cultivars but noted that only 57% of the phenotype observed in the head could be explained by the phenotype seen at the seedling stage. In the present study, QTL associated with both seedling and head blast were observed on chromosome 2B in the BR 18 × BRS 179 population, at 83.8 cM (peak marker at 616,934,668 bp in the reference cv. Chinese Spring sequence) and 98.8 cM (peak marker at 703,976,058 bp) respectively. Whilst the confidence intervals for the two QTL partially overlap, the position of the peak markers suggests they may be separate QTL as the mapping distances correspond to a physical distance of 87.0 Mb between peak markers in the reference cv. Chinese Spring. The *Rmg8* R-gene is also located on chromosome 2B of hexaploid wheat [10], however it has been mapped to the distal region of the long arm of 2B suggesting it is unlikely to represent the QTL seen in this study.

Two seedling QTL were also identified on chromosome 4B. The QTL *QSdl.jic-4B.1* and *QSdl.jic-4B.2* were identified at 129.4 cM (peak marker at 621,326,999 bp) and 127.4 cM (peak marker at 650,634,242 bp) in the Anahuac 75 × BR 18 and BR 18 × BRS 179 populations, respectively. Somewhat surprisingly, while BR 18 contributed the resistant allele in the Anahuac 75 × BR 18 population, it contributed the susceptible allele in the BR 18 × BRS 179 population. It is possible that the genes underlying this seedling-specific resistance form an allelic series or that independent but closely linked genes account for the difference in the contribution to resistance by BR 18 in the two populations. We also identified QTL associated with seedling and heading stage resistance on the long arm of chromosome 5A in both populations. BR 18 conferred the susceptible allele for a seedling QTL in the BR 18 × BRS 179 population at 233.5 cM (peak marker at 678,229,352 bp), whilst Anahuac 75 contributed the susceptible allele for a heading stage QTL in the Anahuac 75 × BR 18 population at 216.5 cM (peak marker at 595,145,761 bp). As there is a physical distance of 83.0 Mb between the peak QTL markers in the reference cv. Chinese Spring, it is unlikely that these are the same QTL. However, it is possible that the differential resistance contributed by BR 18 alleles on chromosome arm 5AL may be due to the presence of closely linked genes associated with seedling and heading stage resistance which are found within this region. Additional mapping studies using other wheat populations will determine whether these regions have an important association with blast resistance.

In the wheat–rust pathosystem, seedling resistance provides resistance at all developmental stages and is often race specific [29]. Seedling resistance is conferred by major *R* genes, which often encode nucleotide-binding site leucine-rich repeat (NBS-LRR) domain proteins. Resistance expressed in the later developmental stages is classified as adult plant resistance (APR). *Lr34*, one of the best characterised APR genes, confers resistance in seedlings only at temperatures below 8 °C, yet provides durable, partial resistance to rust in adult plants [30]. In the rice blast pathosystem, resistance conferred at the leaf and panicle stages appears to differ amongst *R* genes. The NBS-LRR *Pb1* confers blast resistance only in the panicle and is therefore considered to be an APR gene [31], whilst *Pi64*, which also encodes an NBS-LRR, confers resistance to MoO isolates in both the leaf and panicle [32]. In our study we identified several QTL which were specific to a particular developmental stage, such as the seedling-specific QTL on 4B. However, there was also a moderate positive correlation between blast disease scores at the seedling and heading stage, an observation also seen in a panel of U.S. wheat cultivars [17], which may indicate that some seedling resistance loci do have an effect at the head stage. It is possible that the effect of these loci is masked by other genes which are expressed during the later stages of plant development and confer resistance only in the head. However, as the major seedling resistance QTL *QSdl.jic-4B.2* (24.8% var) was not identified at the heading stage it appears less likely that loci conferring more potent seedling resistance also provide resistance in the head. The histograms of the disease phenotype scores indicate a greater level of susceptibility in the head assays when compared to the seedling assays (Figs. 2 and 4), therefore also suggesting that there may be seedling-specific resistance genes. Our study therefore provides genetic evidence to suggest that resistance to blast at the seedling and heading stage is likely to be controlled by different genes, a finding which has important implications when breeding wheat for blast resistance. Whilst the development of blast during anthesis is considered to be the most destructive due to the effect this has on grain production, severe infection during the seedling stage can result in the death of the plant and also provides a source of inoculum for further disease spread. To ensure that resistance is provided at both of these significant stages of plant development, our results suggest that genes which confer resistance at the vegetative and reproductive stages may need to be combined into a cultivar, a process which will require the development of genetic markers to follow the presence of beneficial alleles through a breeding programme.

### Associations between wheat blast and powdery mildew

Whilst few papers have identified QTL associated with blast in wheat, a recent study in barley identified two QTL, on chromosomes 1H and 7H, using the isolate MoT BR32 [20]. In the same study, several blast QTL co-localised with known powdery mildew resistance genes, such as the 7H QTL which mapped within 5 cM of the resistance gene *mlt*. In our study, inoculation with the MoT BR32 isolate did not result in the identification of any QTL associated with blast on chromosomes 7A, 7B or 7D, suggesting it is unlikely there is an association with a wheat orthologue of *mlt* within this region. In barley, Aghnoum et al. [20] did not see an association with blast and mildew on 1H using the BR32 isolate, but the authors did observe the co-localisation of blast QTL with the mildew resistance locus *Mla6* using the MoO Guy11 isolate. The gene *RMo1*, which confers nearly complete resistance to the MoO Ken 54–20 isolate, also co-segregates with the *Mla* locus on the short arm of 1H in barley [33]. Two wheat orthologues of the *Mla* locus are *Sr33* and *Sr50*, which provide stem rust resistance and are located on the short arm of chromosome 1D [34]. Whilst we did not identify any QTL on 1D in our study, *QHead.jic-1A* was identified on chromosome arm 1AS in a region homeologous to *Sr33.* In natural environment conditions the mildew susceptibility of the Anahuac 75 × BR 18 and BR 18 × BRS 179 populations was assessed over two years and no association with powdery mildew on 1A or 1D was observed (Unpublished observations, R Goddard). As*Sr33* is not known to be associated with mildew resistance in wheat, it is unlikely there is a relationship between wheat blast and mildew resistance on 1A.

### Resistance in BR 18-Terena is quantitative

In this study nine QTL were associated with wheat blast. The largest phenotypic effect conferred by these QTL was 24.8%, suggesting that the resistance seen in BR 18 is quantitative. Whilst *R* genes commonly encode NBS-LRR domain proteins, the genes underlying quantitative resistance are more varied, with roles in defence signalling, basal defence mechanisms and toxin detoxification [35]. The APR gene *Lr34* encodes an ATP-binding cassette (ABC) transporter which provides durable resistance to several plant pathogens and has been shown to induce genes regulated by abscisic acid (ABA), a phytohormone with a key role in plant–pathogen interactions [36]. A further APR gene *Yr36*, which encodes *Wheat Kinase START1* (*WKS1*), confers resistance to wheat stripe rust (*Puccinia striiformis* f. sp. *tritici*) by inducing the production of chlorosis and reactive oxygen species (ROS) through protein phosphorylation [37]. Interestingly, a recent study demonstrated that BR 18 had a greater activation of basal resistance mechanisms following MoT infection, compared to the susceptible cultivar BRS Guamirim [38]. The production of defence enzymes such as phenylalanine ammonia-lyase (PAL) and polyphenoloxidase (PPO) was greater in BR 18 flag leaves, as was the production of the antioxidant enzyme superoxide dismutase (SOD), which protects cells from excess ROS produced by the plant following pathogen infection [38]. It is therefore possible that some of the genes underlying the QTL identified within the BR 18 background may play a role in basal resistance responses.

Quantitative resistance is generally thought to be more durable than *R* gene mediated resistance, as it is less likely to be overcome by a changing pathogen population [39], which may explain why BR 18 shows resistance across several environments and with different *M. oryzae* isolates [6, 22]. In this study, BR 18 provided resistance at the heading stage for three QTL on 2B, 4A, 5A. From the panel of Brazilian wheat cultivars also analysed, 11.0% of lines displayed a BR 18-like haplotype at more than one QTL region. Several of these lines such as Quartzo, Fundacep Raizes and TBIO Sinuelo, a cultivar which possesses a BR 18-like haplotype for all three QTL regions, have also been demonstrated to display moderate resistance to blast at the heading stage in field conditions in the Cerrado region of Brazil [40]. This suggests that selecting for the BR 18 haplotype within the QTL regions on 2A, 4B and 5A may provide a basal level of resistance to wheat blast at the heading stage, which could be further enhanced by introducing race-specific resistances. Some studies have observed that under certain conditions BR 18 may display moderate susceptibility [24, 41]. In this study, BR 18 conferred the resistant allele for six of the nine QTL identified, demonstrating that BR 18 has both positive and negative alleles associated with blast resistance. As both the highly susceptible Anahuac 75 and the moderately resistant BRS 179 cultivar also contributed alleles for resistance, this suggests that the resistance of BR 18 can be further improved. In addition, transgressive segregation was observed in both populations, for both seedling and heading stage resistance, demonstrating that the resistance of the parental lines could be increased by combining different alleles in the progeny. Interestingly, the resistance in both populations is quantitatively inherited, however the phenotype of several RILs indicates near immunity to the MoT BR32 isolate. This is reminiscent of a study in barley, where the complete resistance of accession CGN02857 was expected to be monogenically inherited yet polygenic resistance was observed [20]. This suggests that individual accessions of both barley and wheat may exhibit a qualitative phenotype in response to *M. oryzae* infection yet demonstrate quantitative resistance.

### Developing stable wheat blast resistance

Identifying both quantitative resistances and *R* genes which may be introgressed into a single cultivar is particularly important to help control disease in countries such as Brazil, where the MoT pathogen population is diverse and well established [24]. In rice, the stacking of three minor resistance QTL and the major *R* genes *Pi-ta* and *Pi-b* has been shown to have a positive additive effect on blast resistance to several isolates [42]. This demonstrates the advantages of combining both quantitative and *R* gene mediated resistance provided that the epistatic interactions between resistances are understood. In wheat, the newly identified blast resistance gene *RmgGR119* has been demonstrated to act in an additive manner with *Rmg8* to increase resistance against the Brazilian BR48 isolate [43]. This suggests that it may also be possible to combine quantitative resistances, such as those seen within BR 18, with known *R* genes like *Rmg8* and *RmgGR119* to produce wheat cultivars with both race-specific and broad-spectrum resistance. As several *R* genes, such as *Rmg2* and *Rmg3,* and the 2NS translocation have already been shown to be less effective against recent *M. oryzae* isolates [1, 15], combining sources of resistance will be essential to prevent further severe disease outbreaks. Interestingly, as the *M. oryzae* strains isolated from infected wheat in Bangladesh in 2016 were found to be most closely related to the Brazilian isolates PY0925 and BR32 [4], the BR 18 resistance QTL identified within this study could also be of potential use in Bangladesh. Additional trials in Bangladesh with BR 18 and other Brazilian cultivars with stable resistance would be required to determine whether these resistances are suitable for that specific environment.

## Conclusions

This study presents the first investigation into wheat blast resistance from the BR 18 genetic background. As BR 18 has been widely used in Brazilian breeding programmes for several decades, not only due to stable blast resistance but also advantageous quality traits and an ability to grow under poor environmental conditions [44], it is particularly important to understand the genetics behind these favourable characteristics. We identified a total of nine QTL associated with blast, suggesting the resistance observed is quantitative, with BR 18 providing the resistant allele at six of these loci. Importantly, we also identified that seedling and head blast resistance do not appear to be governed by the same loci, which has implications when breeding wheat to be blast resistant at different developmental stages. Identification of genomic regions associated with blast resistance, both in BR 18 and other cultivars with stable resistance, should allow genetic markers to be developed to both track and combine these specific resistances within breeding programmes. Refining the genomic regions associated with blast resistance will be important in order to develop genetic markers which are closely linked to the trait of interest and are suitable for high-throughput genotyping. It will also be crucial to identify whether blast resistance loci have any pleiotropic effects on yield, grain quality or disease resistance in order to determine which resistances are appropriate for selection in breeding programmes. As more QTL studies are undertaken it will also become apparent which loci provide novel resistance and which loci have already been introgressed into existing wheat cultivars. This information should ultimately provide wheat breeders with more comprehensive knowledge of the available resistances to effectively control wheat blast disease.

## Methods

### Plant material

Seed of the spring wheat varieties BR 18-Terena (unknown pedigree) [44], Anahuac 75 (I-12300//Lerma-Rojo-64/II-8156/3/Norteno-67) [44] and BRS 179 (BR 35/PF 8596/3/PF 772003*2/PF 813//PF 83899) [44] were provided by Embrapa Wheat, Passo Fundo, Rio Grande do Sul, Brazil. Anahuac 75 is known to be susceptible to blast [22], whilst BRS 179 is moderately resistant/susceptible [45]. BR 18-Terena was used as a common parent in the development of two bi-parental crosses by single seed descent: Anahuac 75 × BR 18 and BR 18 × BRS 179. For both populations the last single seed selection was made at the F_6_ generation, and a total of 188 recombinant inbred lines (RILs) were developed for each cross.

### Fungal inoculum

The MoT pathotype isolate BR32, which has been fully sequenced [27], was selected from the JIC culture collection and maintained at 25 °C [46]. For each experiment, inoculum was produced from filter paper stocks of *M. oryzae* BR32 mycelium to maintain the virulence of the isolate. Briefly, filter paper stocks were grown on complete media agar (CMA) for 14 days at 22 °C. The initial plates were then sub-cultured and grown for an additional 14 days before conidia were harvested. Conidial inoculum was prepared by washing the culture plates with 6 ml of ddH_2_O and using a glass rod to remove the conidia. The resulting conidial suspension was filtered through two layers of cheesecloth and the conidia density was counted using a haemocytometer. For both seedling and head assays the conidial suspension was adjusted to 0.3–0.4 × 10^6^ conidia per ml.

### Wheat blast phenotyping

Seedling resistance to MoT was assessed using detached leaf assays, using the method of Chen et al. [47]. Seeds of the F_6_ RILs and the parental lines were incubated for 48 h in the dark at 4 °C in 9 cm Petri dishes containing filter paper and 4 ml of 2 μM GA_3_ (Sigma Aldrich). Seeds were transferred to 20 °C for 24 h and then planted in 96- cell trays, with a single seed per tray, in peat-based compost. Seedlings were grown to the 2nd leaf stage at 18/15 °C under a 16 h/8 h light-dark photoperiod in a controlled environment room (CER). The second leaf of each seedling was detached, cut into an 8 cm section and placed, adaxial side up, between strips of 1% water agar in 10 × 10 cm clear plastic plates. Six RIL genotypes were assayed per plate, with a single leaf per genotype and all plates contained a susceptible control. Due to seed availability, Anahuac 75 was used as the susceptible control in the Anahuac 75 × BR 18 assays, whilst Hobbit-sib (Dwarf A) was used in the BR 18 × BRS 179 assays. Per genotype, five replicate leaves were inoculated. Leaves were spray inoculated with BR32 conidial suspension using an air brush sprayer, at a volume of 25 ml per 15 plates. The lids of the plates were misted with H_2_O to increase humidity and plates were laid flat in plastic trays, inside a clear plastic cover. Trays were kept in the dark for 24 h after inoculation and incubated at 24 °C under a 16 h/8 h light-dark photoperiod. At 6 days post inoculation [dpi] leaves were scored for disease symptoms using a 0–6 scale (0 = no visible symptoms, 1 = pin-point brown necrotic lesions, 2 = brown necrotic lesions across the leaf, 3 = brown necrotic lesions and mild chlorosis of the leaf, 4 = grey lesions ringed with necrosis and chlorosis, 5 = extensive grey lesions and chlorosis across the leaf, 6 = grey sporulating lesions and water soaking across the entire leaf). A representative scoring scale can be seen in Additional File 11.

Resistance in the head was assessed using detached head assays. Per population, 100 lines tested at the seedling stage and each of the parents were assayed. Plants were grown in 2 L pots, containing a peat-based medium, with three plants per genotype per pot, in a glasshouse at 18/15 °C under a 16 h/8 h light-dark photoperiod. At Zadoks growth stage 61 (GS61) [48] heads were detached from the stem between the penultimate and final nodes. The stem was cut again, under water, above the final node and then placed upright in 200 μl plastic pipette tip boxes filled with H_2_O. A total of 12 detached heads were included per box. A minimum of three replicates, from three different plants, were inoculated per genotype. Heads were sprayed until run-off with BR32 conidial suspension, and boxes were placed in propagation trays and covered with plastic lids. Trays were kept in the dark for 24 h after inoculation and misted with H_2_O following inoculation to increase the humidity and promote fungal development. Boxes were incubated at 24 °C under a 16 h/8 h light-dark photoperiod. At 9 dpi heads were scored for disease symptoms using a 0–6 scale (0 = no visible symptoms, 1 = pin-point brown lesions, 2 = small brown lesions, 3 = brown lesions, very small areas of bleaching on glumes, 4 = few brown lesions, bleaching of glumes, 5 = bleaching of glumes, 6 = complete bleaching of the head). A representative scoring scale can be seen in Additional File 12.

### Genotyping of the bi-parental populations

For both populations leaf material from 3-week old seedlings was sampled, with a pool of six seedlings sampled per genotype. Genomic DNA was extracted using the protocol described by Pallotta et al. [49]. The Axiom® 35 k Wheat Breeders’ Array [28] was used to genotype both populations using the Affymetrix GeneTitan® analyser in a 384-sample format (Bristol Genomics Facility, Bristol University, Bristol). Data calling was performed using the Axiom® Affymetrix Analysis Suite (version 2.0.0.35) using the ‘Axiom® Best Practices Genotyping Workflow’ for hexaploid wheat. For both populations, monomorphic markers and markers with missing genotype data for the parental lines were removed from the analysis. Genotype calls for polymorphic markers are presented in Additional File 13. Genetic linkage groups for each population were created using the online version of MST map [50]. Markers with over 20% missing data were omitted from the analysis, and a mapping size threshold of two and a mapping distance threshold of 15 cM were required for linkage. The Kosambi mapping function was used to calculate genetic distances between markers. Genetically redundant markers were removed from the final linkage groups and the marker order for each chromosome was ordered according to the IWGSC RefSeq v1.0 [51] wheat genome assemblies for cv. Chinese Spring.

### Statistical and QTL analysis

Analyses of variance [ANOVA] for phenotypic traits were conducted by means of a general linear model (GLM) within Genstat 20th edition [52]. For both seedling and head assays, plate/box, replicate and genotype were included as model terms. To determine the association between seedling and head blast experiments, Pearson’s correlation coefficients were calculated. Predicted mean values for each RIL were calculated for all datasets within each GLM and were used for QTL analysis. QTL analysis was performed in Genstat using single-trait, single-environment analysis. For all analyses, a logarithm of the odds (LOD) score of 3.0 was required for a QTL to be deemed significant and a mapping step size of 5 cM was used. Initial QTL detection was performed using simple interval mapping (SIM), followed by at least two rounds of composite interval mapping (CIM) to finalise the QTL location using the candidate QTL as co-factors. A final QTL model was then fitted to produce the estimated QTL effects and QTL names were assigned using recommended rules for QTL naming. QTL images were produced using MapChart [53].

### Haplotype analysis of Brazilian wheat cultivars

Seed of 100 Brazilian wheat cultivars was provided by Embrapa Wheat, Passo Fundo, Rio Grande do Sul, Brazil (Additional File 9) and DNA was extracted using a CTAB method [54]. The panel was genotyped using the wheat 90 K Infinium iSelect assay [55] at the Bristol Genomics Facility (Bristol University, Bristol) with single nucleotide polymorphism (SNP) calling performed using the methods as described by Wang et al. [55] to give raw data for 81,587 SNPs. SNPs were aligned to the IWGSC RefSeq v1.0 assembly [51] and sorted by their chromosome position. Heterozygous SNP calls were classified as missing data and SNPs with over 5.0% missing data were removed. For the identified heading stage QTL, the physical position of the QTL flanking markers at the LOD 3 threshold was determined. iSelect markers which mapped within the QTL intervals were identified and the genotype calls of the Brazilian cultivar panel were compared to those of the resistant parent for each marker within each region (Additional File 9). Cultivars were classified as having a ‘resistant parent-like’ haplotype if they displayed 98.0% identical genotype calls to the resistant parent for markers within the specified QTL region.

## Supplementary information

**Additional file 1: Figure S1.** QTL identified on 1A in the Anahuac 75 × BR 18 F_6_ RIL population.

**Additional file 2: Figure S2.** QTL identified on 4A in the Anahuac 75 × BR 18 F_6_ RIL population.

**Additional file 3: Figure S3.** QTL identified on 4B in the Anahuac 75 × BR 18 F_6_ RIL population.

**Additional file 4: Figure S4.** QTL identified on 5A in the Anahuac 75 × BR 18 F_6_ RIL population.

**Additional file 5: Figure S5.** QTL identified on 6A in the Anahuac 75 × BR 18 F_6_ RIL population.

**Additional file 6: Figure S6.** QTL identified on 2B in the BR 18 × BRS 179 F_6_ RIL population.

**Additional file 7: Figure S7.** QTL identified on 4B in the BR 18 × BRS 179 F_6_ RIL population.

**Additional file 8: Figure S8.** QTL identified on 5A in the BR 18 × BRS 179 F_6_ RIL population.

**Additional file 9.** Haplotype data for 100 Brazilian cultivars at the 1A, 2B, 4A and 5A QTL regions.

**Additional file 10: Table S1.** Cultivars displaying a 98.0% similar haplotype to Anahuac 75 at the 1A head resistance QTL and to BR 18-Terena at the 2B, 4A and 5A head resistance QTL.

**Additional file 11: Figure S9.** Representative detached leaf assay 0–6 scoring scale. Scale bar indicates 1 cm.

**Additional file 12: Figure S10.** Representative detached head assay 0–6 scoring scale. Scale bar indicates 1 cm.

**Additional file 13.** Genotype and phenotype datasets for both the Anahuac 75 × BR 18 and BR 18 × BRS 179 F_6_ RIL populations.

## Data Availability

All data generated or analysed during this study are included in this published article and its supplementary information files.

## Abbreviations

GLM: General linear model
MoO: *Magnaporthe oryzae Oryza* pathotype
MoT: *Magnaporthe oryzae Triticum* pathotype
QTL: Quantitative trait loci
RIL: Recombinant inbred line
SNP: Single nucleotide polymorphism
