# Supplementary figures and images for: Dissecting the genetic basis of wheat blast resistance in the Brazilian wheat cultivar BR 18-Terena
Source: BMC Plant Biol. 2020 Aug 27;20:398. doi: 10.1186/s12870-020-02592-0 (PMC7451118; doi:10.1186/s12870-020-02592-0)

## Slide 1
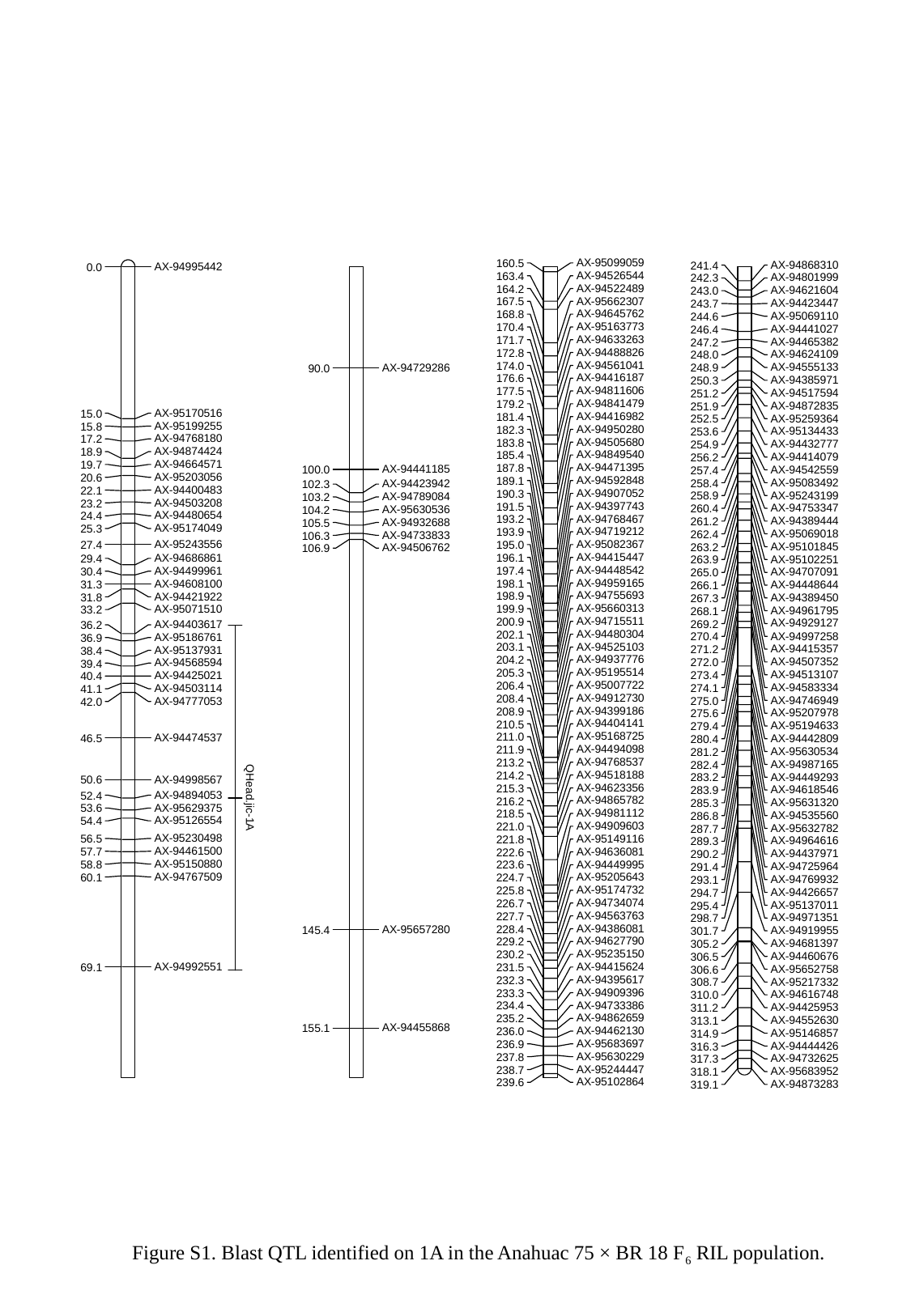

Figure S1. Blast QTL identified on 1A in the Anahuac 75 × BR 18 F6 RIL population.

Supplement: Supplementary file 1 — Additional file 1: Figure S1. QTL identified on 1A in the Anahuac 75 × BR 18 F6 RIL population. [file 12870_2020_2592_MOESM1_ESM.pptx]

## Slide 1
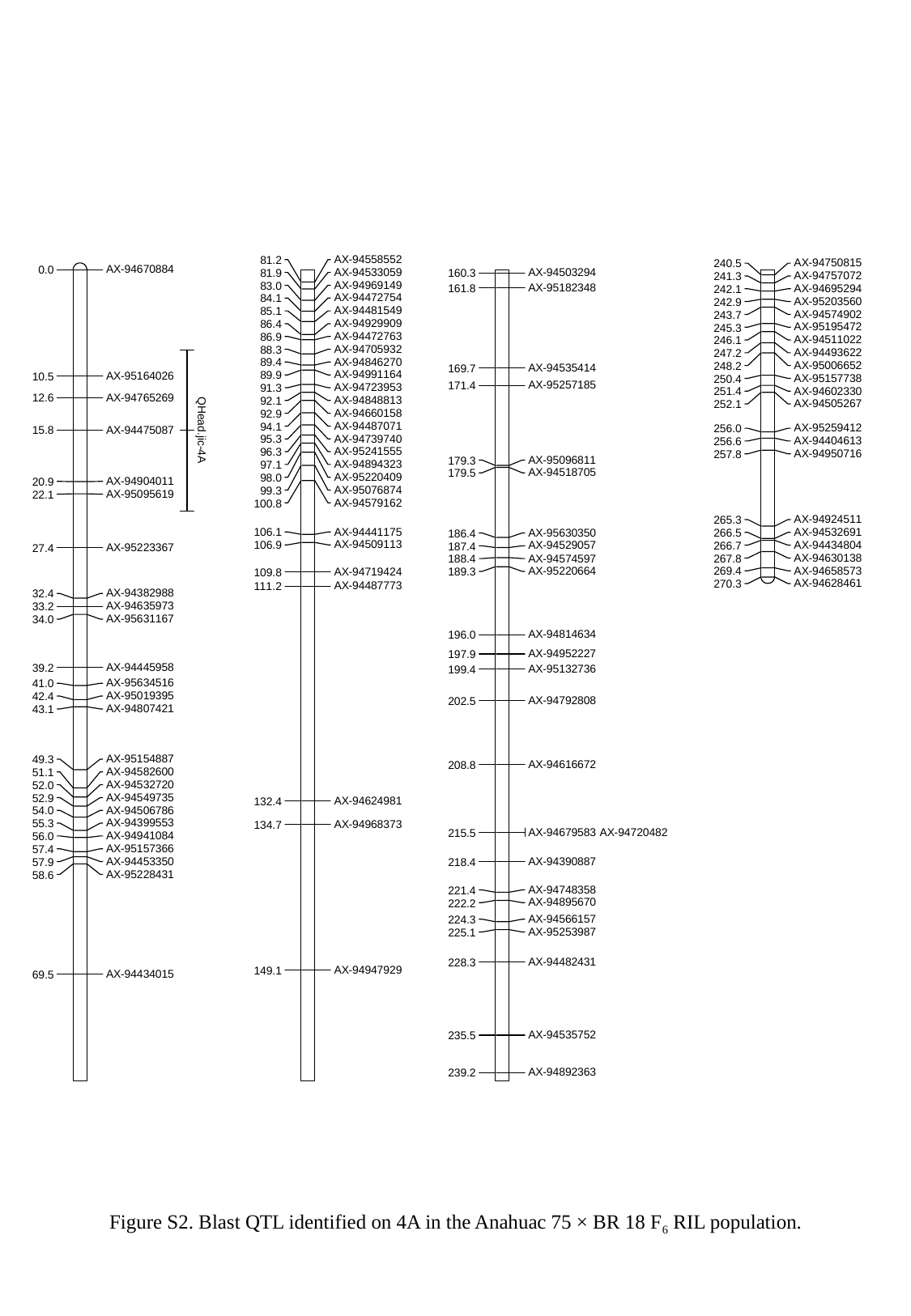

Figure S2. Blast QTL identified on 4A in the Anahuac 75 × BR 18 F6 RIL population.

Supplement: Supplementary file 2 — Additional file 2: Figure S2. QTL identified on 4A in the Anahuac 75 × BR 18 F6 RIL population. [file 12870_2020_2592_MOESM2_ESM.pptx]

## Slide 1
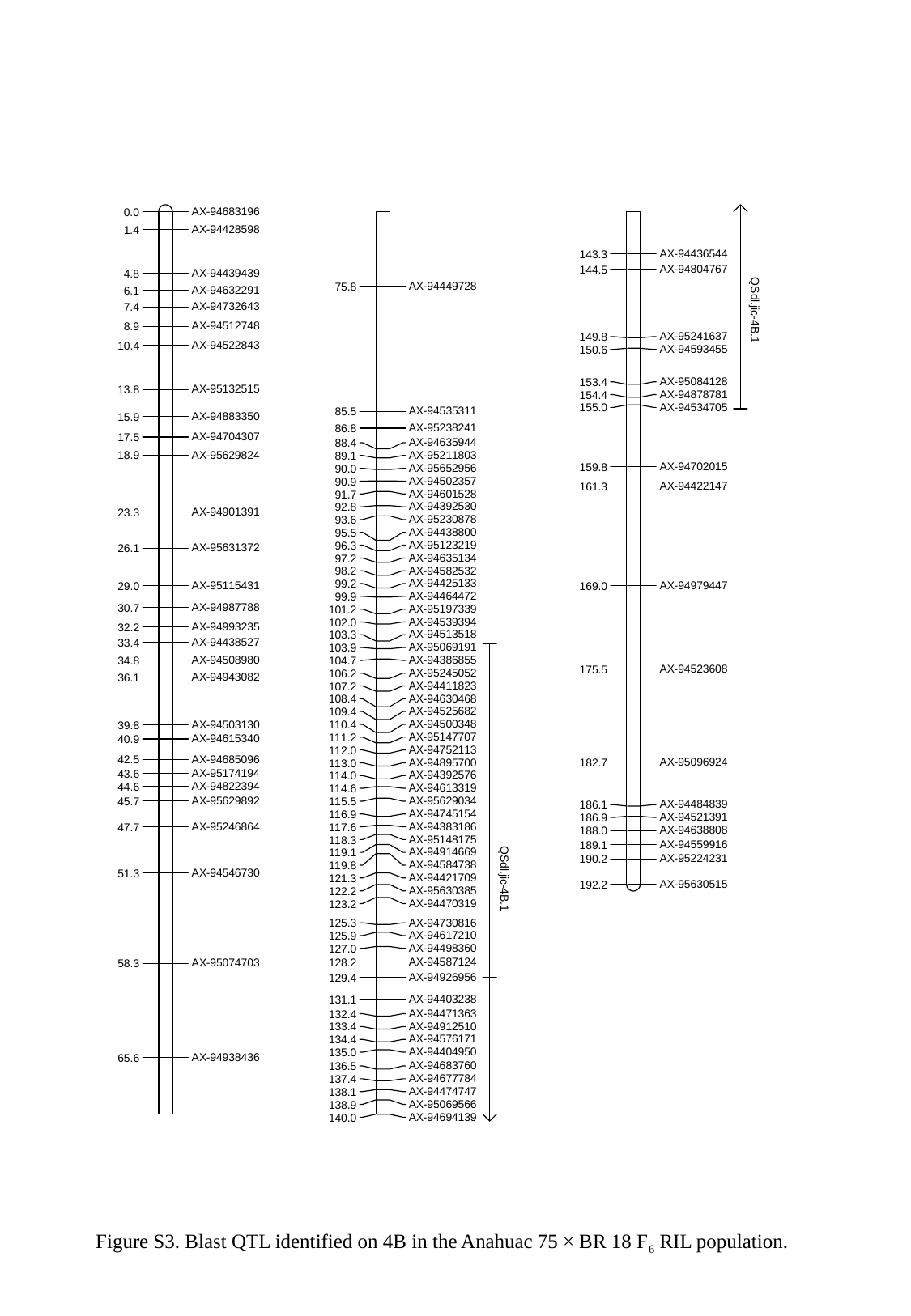

Figure S3. Blast QTL identified on 4B in the Anahuac 75 × BR 18 F6 RIL population.

Supplement: Supplementary file 3 — Additional file 3: Figure S3. QTL identified on 4B in the Anahuac 75 × BR 18 F6 RIL population. [file 12870_2020_2592_MOESM3_ESM.pptx]

## Slide 1
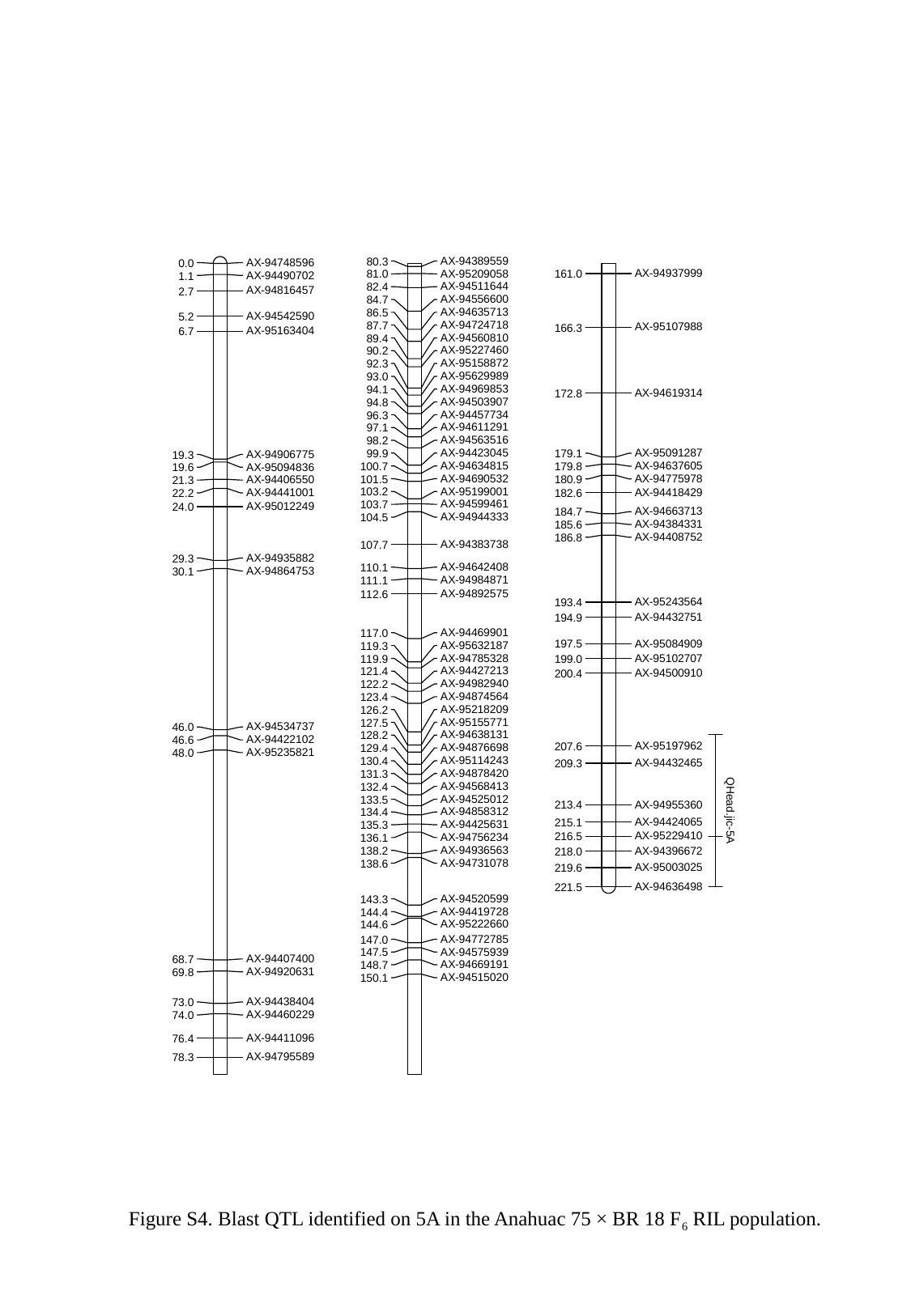

Figure S4. Blast QTL identified on 5A in the Anahuac 75 × BR 18 F6 RIL population.

Supplement: Supplementary file 4 — Additional file 4: Figure S4. QTL identified on 5A in the Anahuac 75 × BR 18 F6 RIL population. [file 12870_2020_2592_MOESM4_ESM.pptx]

## Slide 1
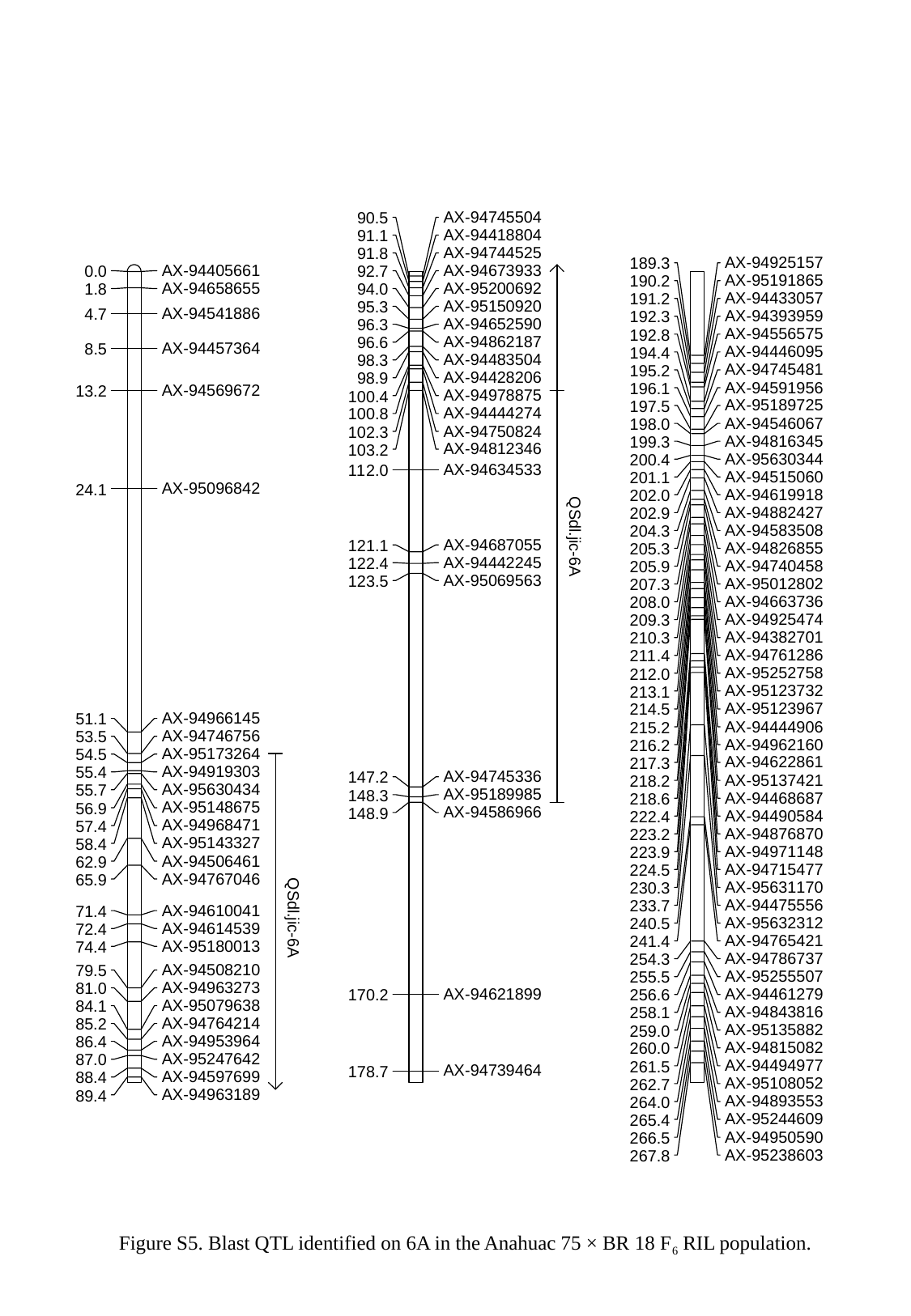

Figure S5. Blast QTL identified on 6A in the Anahuac 75 × BR 18 F6 RIL population.

Supplement: Supplementary file 5 — Additional file 5: Figure S5. QTL identified on 6A in the Anahuac 75 × BR 18 F6 RIL population. [file 12870_2020_2592_MOESM5_ESM.pptx]

## Slide 1
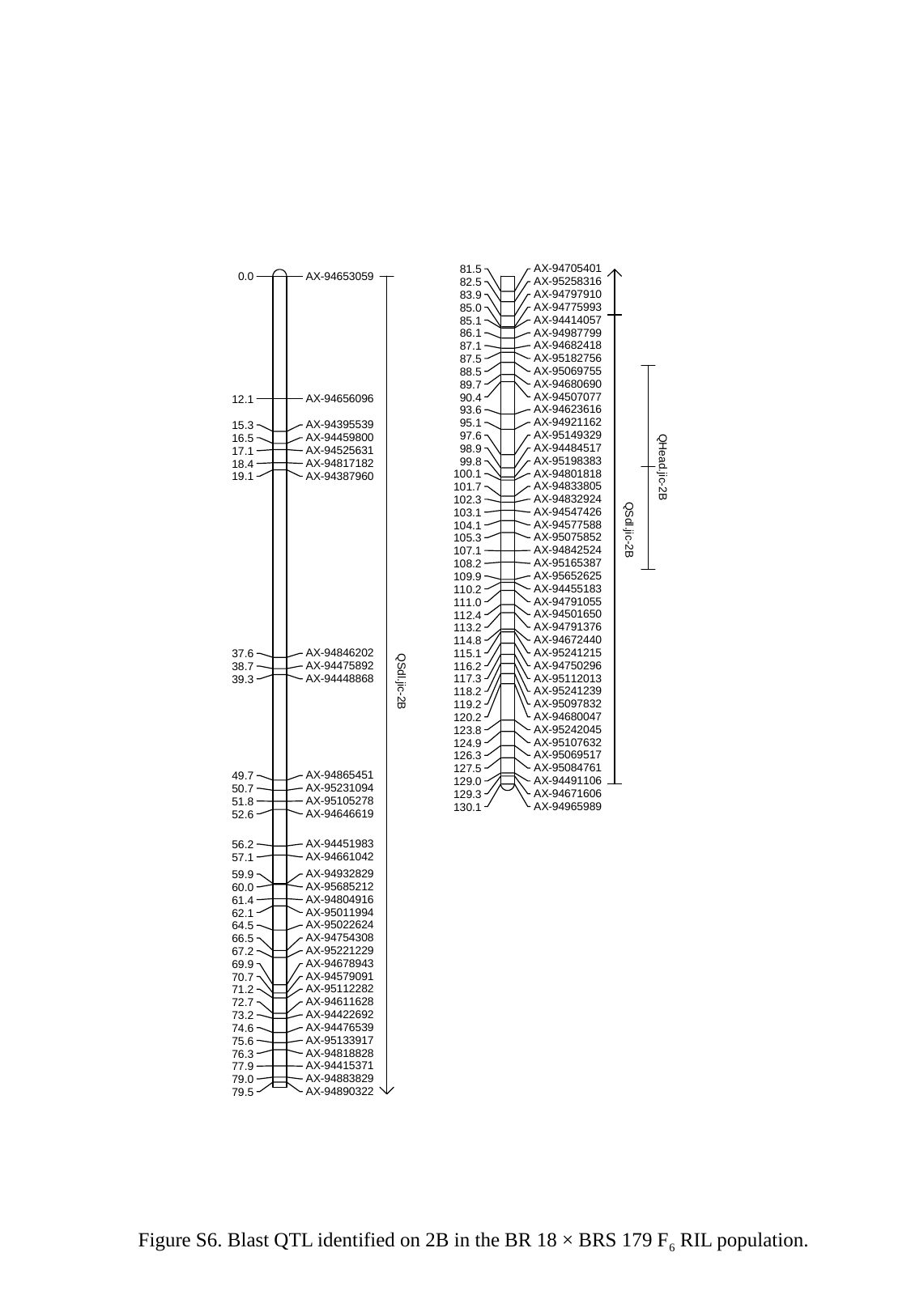

Figure S6. Blast QTL identified on 2B in the BR 18 × BRS 179 F6 RIL population.

Supplement: Supplementary file 6 — Additional file 6: Figure S6. QTL identified on 2B in the BR 18 × BRS 179 F6 RIL population. [file 12870_2020_2592_MOESM6_ESM.pptx]

## Slide 1
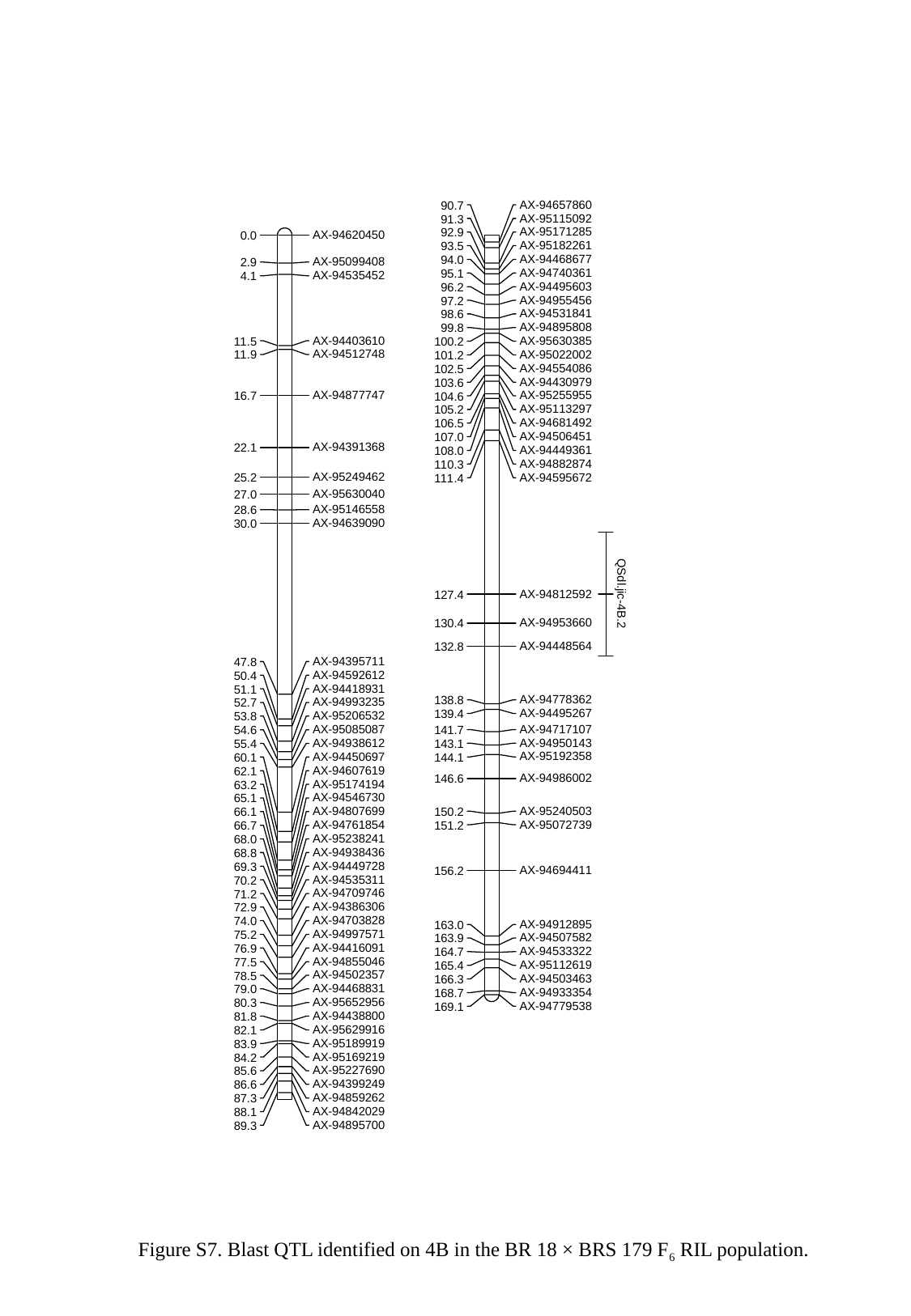

Figure S7. Blast QTL identified on 4B in the BR 18 × BRS 179 F6 RIL population.

Supplement: Supplementary file 7 — Additional file 7: Figure S7. QTL identified on 4B in the BR 18 × BRS 179 F6 RIL population. [file 12870_2020_2592_MOESM7_ESM.pptx]

## Slide 1
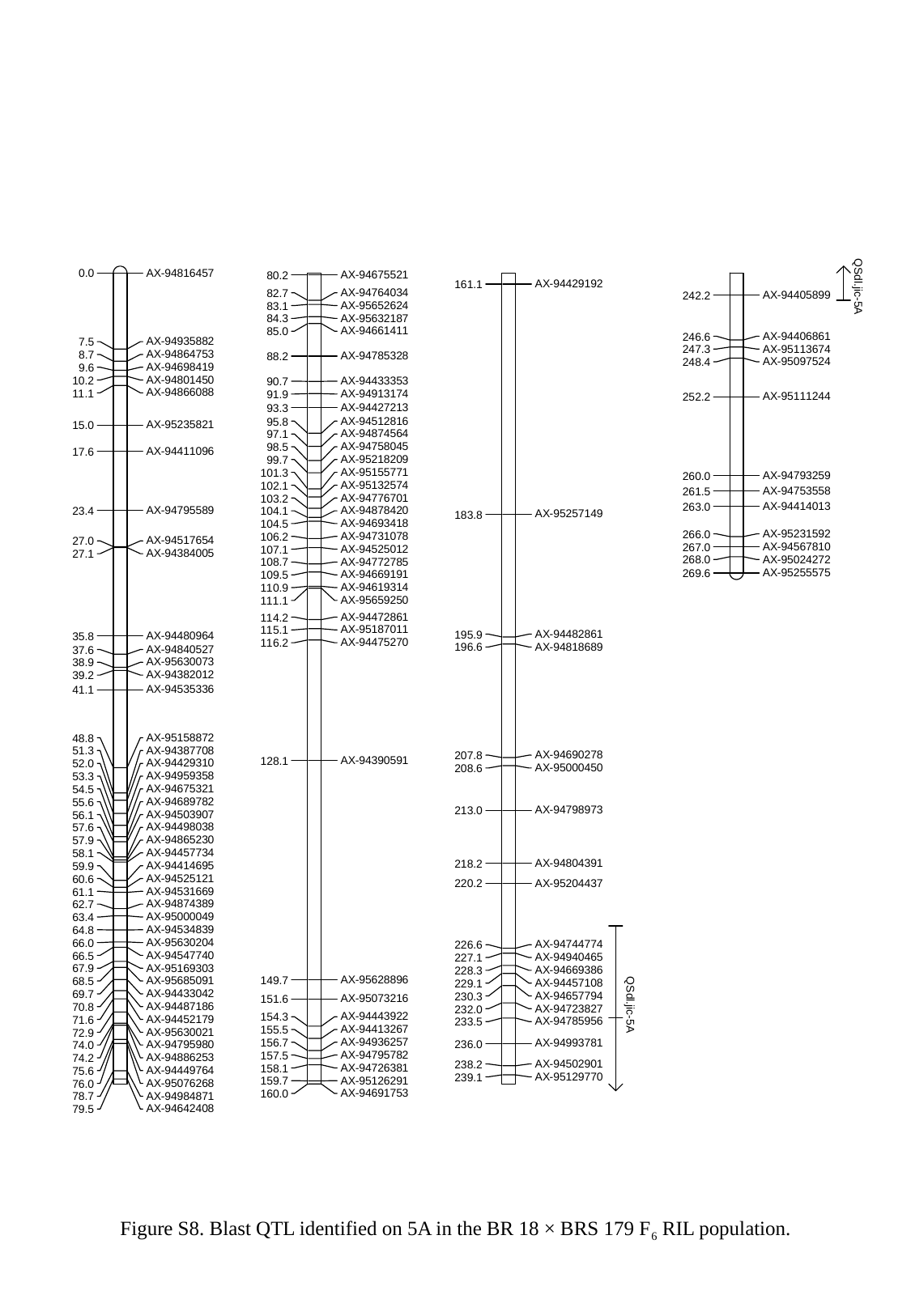

Figure S8. Blast QTL identified on 5A in the BR 18 × BRS 179 F6 RIL population.

Supplement: Supplementary file 8 — Additional file 8: Figure S8. QTL identified on 5A in the BR 18 × BRS 179 F6 RIL population. [file 12870_2020_2592_MOESM8_ESM.pptx]

## Slide 1
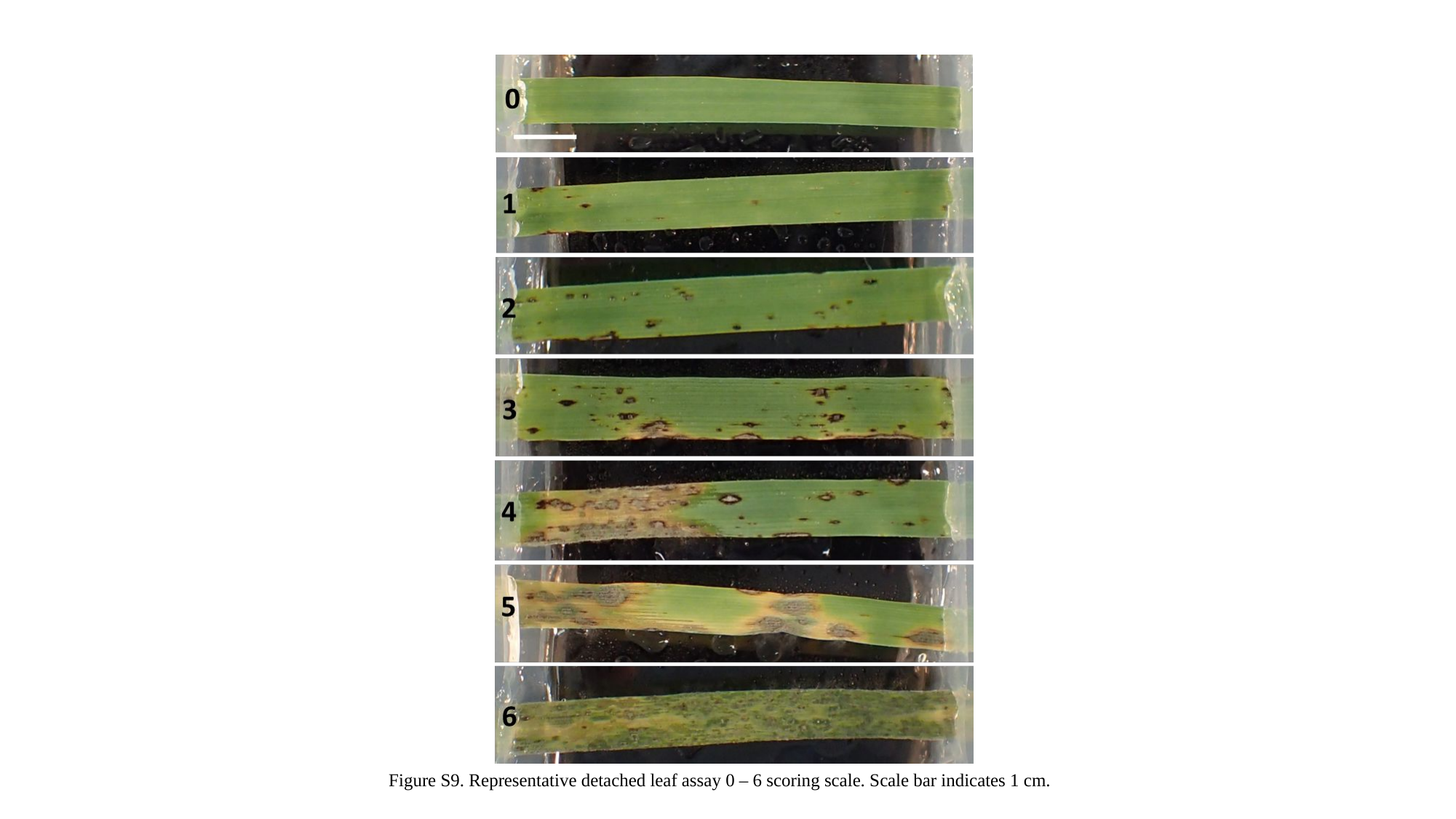

Figure S9. Representative detached leaf assay 0 – 6 scoring scale. Scale bar indicates 1 cm.

Supplement: Supplementary file 11 — Additional file 11: Figure S9. Representative detached leaf assay 0–6 scoring scale. Scale bar indicates 1 cm. [file 12870_2020_2592_MOESM11_ESM.pptx]

## Slide 1
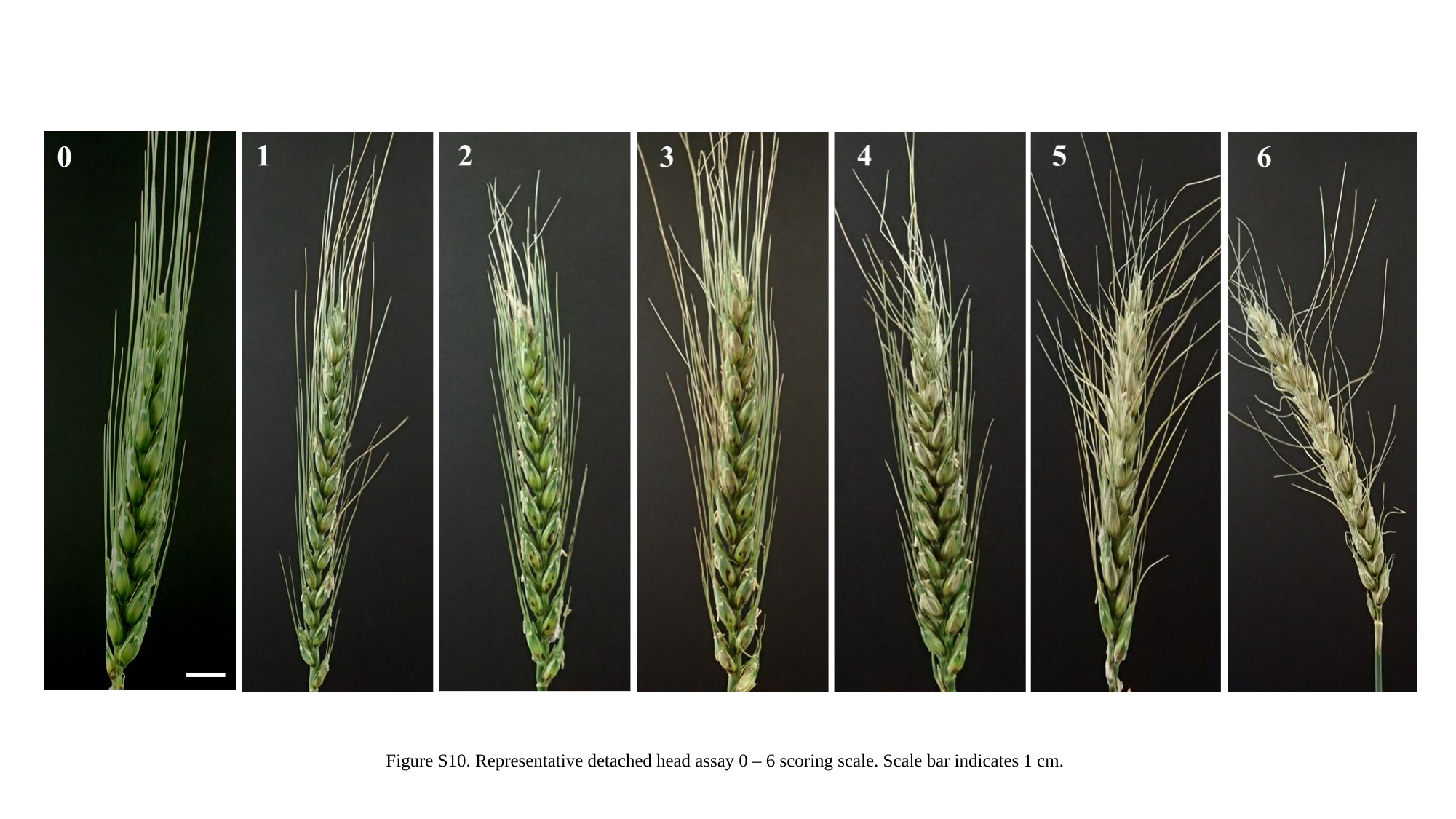

Figure S10. Representative detached head assay 0 – 6 scoring scale. Scale bar indicates 1 cm.

Supplement: Supplementary file 12 — Additional file 12: Figure S10. Representative detached head assay 0–6 scoring scale. Scale bar indicates 1 cm. [file 12870_2020_2592_MOESM12_ESM.pptx]
