# Supplementary material for: Dissecting the genetic basis of wheat blast resistance in the Brazilian wheat cultivar BR 18-Terena
Source: BMC Plant Biol. 2020 Aug 27;20:398. doi: 10.1186/s12870-020-02592-0 (PMC7451118; doi:10.1186/s12870-020-02592-0)
Supplement: Supplementary file 10 — Additional file 10: Table S1. Cultivars displaying a 98.0% similar haplotype to Anahuac 75 at the 1A head resistance QTL and to BR 18-Terena at the 2B, 4A and 5A head resistance QTL. [file 12870_2020_2592_MOESM10_ESM.pptx]

## Slide 1
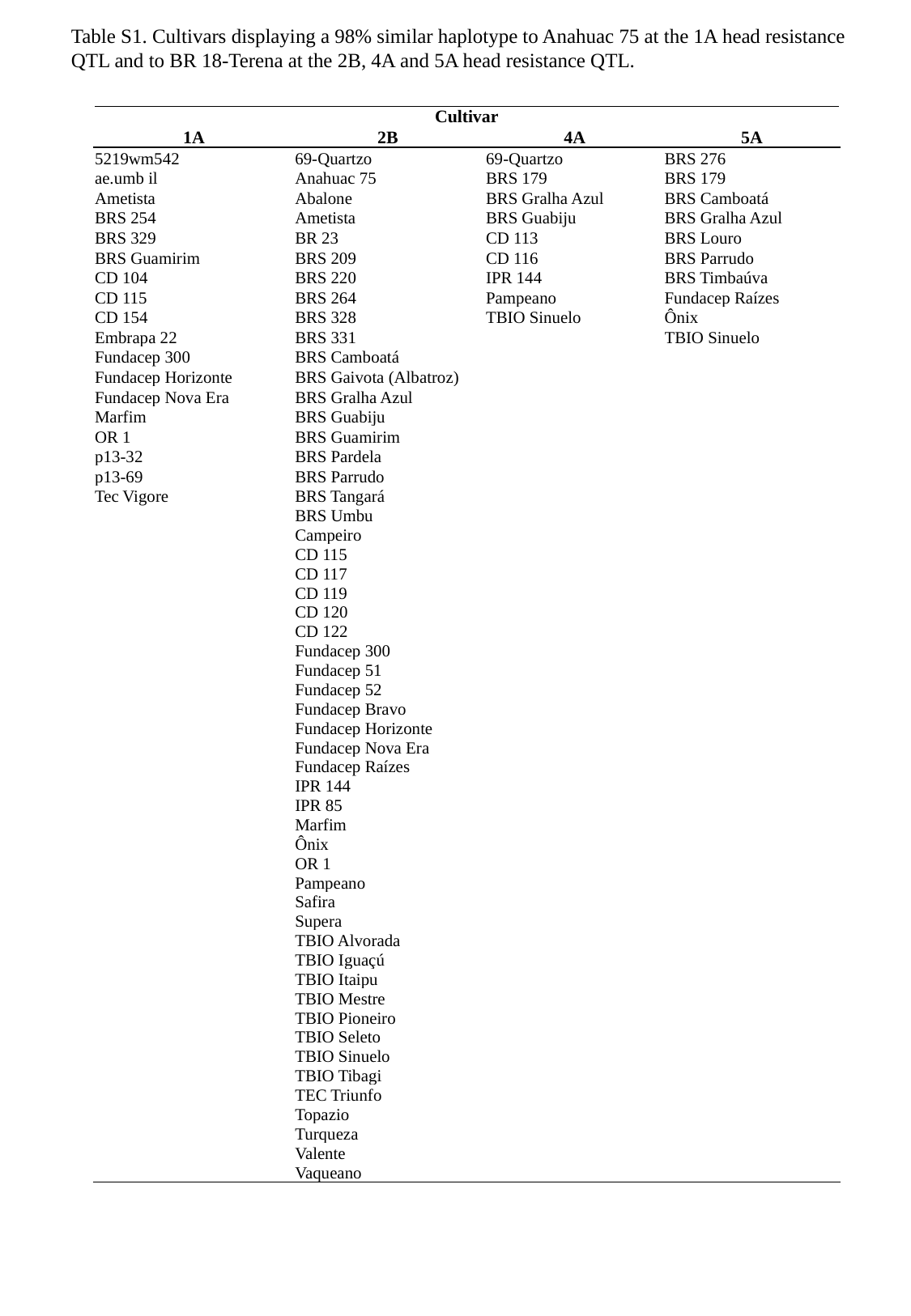

Table S1. Cultivars displaying a 98% similar haplotype to Anahuac 75 at the 1A head resistance QTL and to BR 18-Terena at the 2B, 4A and 5A head resistance QTL.
| Cultivar | | | |
| --- | --- | --- | --- |
| 1A | 2B | 4A | 5A |
| 5219wm542 | 69-Quartzo | 69-Quartzo | BRS 276 |
| ae.umb il | Anahuac 75 | BRS 179 | BRS 179 |
| Ametista | Abalone | BRS Gralha Azul | BRS Camboatá |
| BRS 254 | Ametista | BRS Guabiju | BRS Gralha Azul |
| BRS 329 | BR 23 | CD 113 | BRS Louro |
| BRS Guamirim | BRS 209 | CD 116 | BRS Parrudo |
| CD 104 | BRS 220 | IPR 144 | BRS Timbaúva |
| CD 115 | BRS 264 | Pampeano | Fundacep Raízes |
| CD 154 | BRS 328 | TBIO Sinuelo | Ônix |
| Embrapa 22 | BRS 331 | | TBIO Sinuelo |
| Fundacep 300 | BRS Camboatá | | |
| Fundacep Horizonte | BRS Gaivota (Albatroz) | | |
| Fundacep Nova Era | BRS Gralha Azul | | |
| Marfim | BRS Guabiju | | |
| OR 1 | BRS Guamirim | | |
| p13-32 | BRS Pardela | | |
| p13-69 | BRS Parrudo | | |
| Tec Vigore | BRS Tangará | | |
| | BRS Umbu | | |
| | Campeiro | | |
| | CD 115 | | |
| | CD 117 | | |
| | CD 119 | | |
| | CD 120 | | |
| | CD 122 | | |
| | Fundacep 300 | | |
| | Fundacep 51 | | |
| | Fundacep 52 | | |
| | Fundacep Bravo | | |
| | Fundacep Horizonte | | |
| | Fundacep Nova Era | | |
| | Fundacep Raízes | | |
| | IPR 144 | | |
| | IPR 85 | | |
| | Marfim | | |
| | Ônix | | |
| | OR 1 | | |
| | Pampeano | | |
| | Safira | | |
| | Supera | | |
| | TBIO Alvorada | | |
| | TBIO Iguaçú | | |
| | TBIO Itaipu | | |
| | TBIO Mestre | | |
| | TBIO Pioneiro | | |
| | TBIO Seleto | | |
| | TBIO Sinuelo | | |
| | TBIO Tibagi | | |
| | TEC Triunfo | | |
| | Topazio | | |
| | Turqueza | | |
| | Valente | | |
| | Vaqueano | | |
